# Spatial Distribution of Prominin-1 (CD133) – Positive Cells within Germinative Zones of the Vertebrate Brain

**DOI:** 10.1371/journal.pone.0063457

**Published:** 2013-05-27

**Authors:** József Jászai, Sylvi Graupner, Elly M. Tanaka, Richard H. W. Funk, Wieland B. Huttner, Michael Brand, Denis Corbeil

**Affiliations:** 1 Tissue Engineering Laboratories (BIOTEC), Medical Faculty Carl Gustav Carus, Technische Universität Dresden, Dresden, Germany; 2 DFG Research Center and Cluster of Excellence for Regenerative Therapies Dresden (CRTD), Technische Universität Dresden, Dresden, Germany; 3 Department of Anatomy, Medical Faculty Carl Gustav Carus, Technische Universität Dresden, Dresden, Germany; 4 Max-Planck-Institute of Molecular Cell Biology and Genetics, Dresden, Germany; Duke University Medical Center, United States of America

## Abstract

**Background:**

In mammals, embryonic neural progenitors as well as adult neural stem cells can be prospectively isolated based on the cell surface expression of prominin-1 (CD133), a plasma membrane glycoprotein. In contrast, characterization of neural progenitors in non-mammalian vertebrates endowed with significant constitutive neurogenesis and inherent self-repair ability is hampered by the lack of suitable cell surface markers. Here, we have investigated whether prominin-1–orthologues of the major non-mammalian vertebrate model organisms show any degree of conservation as for their association with neurogenic geminative zones within the central nervous system (CNS) as they do in mammals or associated with activated neural progenitors during provoked neurogenesis in the regenerating CNS.

**Methods:**

We have recently identified prominin-1 orthologues from zebrafish, axolotl and chicken. The spatial distribution of prominin-1–positive cells – in comparison to those of mice – was mapped in the intact brain in these organisms by non-radioactive *in situ* hybridization combined with detection of proliferating neural progenitors, marked either by proliferating cell nuclear antigen or 5-bromo-deoxyuridine. Furthermore, distribution of prominin-1 transcripts was investigated in the regenerating spinal cord of injured axolotl.

**Results:**

Remarkably, a conserved association of prominin-1 with germinative zones of the CNS was uncovered as manifested in a significant co-localization with cell proliferation markers during normal constitutive neurogenesis in all species investigated. Moreover, an enhanced expression of prominin-1 became evident associated with provoked, compensatory neurogenesis during the epimorphic regeneration of the axolotl spinal cord. Interestingly, significant prominin-1–expressing cell populations were also detected at distinct extraventricular (parenchymal) locations in the CNS of all vertebrate species being suggestive of further, non-neurogenic neural function(s).

**Conclusion/Interpretation:**

Collectively, our work provides the first data set describing a comparative analysis of prominin-1–positive progenitor cells across species establishing a framework for further functional characterization in the context of regeneration.

## Introduction

Cellular and molecular characterization of neurogenic niches in the adult vertebrate nervous system is important in elucidating mechanisms underlying endogenous regenerative cascades as well as in elaborating potential cell-based therapeutic approaches. In the adult mammalian telencephalon, there are only two major foci described with constitutive neurogenic activity, which sharply contrasts the widespread embryonic neurogenesis observed along the entire neuraxis [1]–[3]. The significance of this adult phenomenon is not fully understood, but recent findings indicate that it might have an impact among others on spatial memory [4], [5]. Under pathologic conditions (i.e. stroke and traumatic brain injury) the neurogenic activity within the constitutively active foci is markedly enhanced, and to a variable degree, the newly generated cells are recruited to the injury site. The extent of endogenous regenerative processes is nevertheless insufficient to achieve a complete functional recovery [6; reviewed in 7]. Indeed, most of the generated neurons die [6] and a glial scar occurs [8]–[10]. For instance, the probability for recovery of locomotor function is not more than 1% upon complete spinal cord injury [reviewed in 11]. The cellular source of newly generated neuronal cells during both constitutive and injury-induced neurogenesis is apparently a multipotent cell population with phenotypic traits of glial cells [1], [7], [12], [13]. Interestingly, the ependymal cells lining the ventricle system – previously proposed to act as neural stem cells [9]– represent rather a quiescent and/or latent reservoir of neurogenic cells that could be activated in response to injury, transforming to radial glial cells and giving rise to astrocytes and neuroblasts [14]–[17]. The self-renewing ability of these cells in vivo is very likely disabled [16].

In contrast to mammals, cold-blooded (poikilothermic) non-mammalian aquatic vertebrate organisms and, to certain extent, embryonic chick have an intrinsic ability for spontaneous complete regeneration being able to restore complex anatomical structures (epimorphic regeneration), and remarkably, even parts of their central nervous system (CNS) [10. 18–22]. This peculiarity of poikilothermic vertebrates is apparently not independent of their perpetual growth implying that beyond a possible homeostatic replacement/renewal of tissues newly generated cells are routinely added to the already existing ones resulting in net growth.

Interestingly, the CNS of adult non-mammalian vertebrates is characterized by multiple neurogenic foci spread essentially along the entire extent of cerebral ventricular zone [23]–[26]. The analysis of proliferating progenitor and stem cells found therein revealed that they share some key phenotypic and morphologic features with mammalian neural progenitors especially with fetal ones having radial glial morphology [27], [28]. In the brain of adult non-mammalian vertebrates, as a separate pool of ependymal cells does not seem to differentiate from radial glial cells as it does in the mammalian brain, the radial glial cells line directly the ventricular surface [22], [24], [27], [29]. These radial ependymoglial cells are phenotypically heterogeneous as a significant population of them is in a quiescent state depending on their spatial position along the neuraxis [28]. Nevertheless, radial ependymoglial cells of essentially quiescent (i.e. non-germinative) zones of the ventricular surface could be stimulated to re-enter the cell cycle and act as mutipotential progenitors as was exemplified by spinal cord lesion in adult zebrafish [30] or in a toxin-induced lesion of regeneration model of midbrain dopaminergic neurons in metamorphosed newt [31]. Moreover, in the fish brain a relatively large number of quiescent parenchymal oligodendroglial progenitor cells were observed [32].

Comparisons between mammalian and non-mammalian brains are of utmost importance to find some basic lines common to all neurogenic (stem) cells, and to decipher the molecular specificities that allow the impressive neural regenerative capacity of non-mammalian vertebrates. Unfortunately, the prospective identification, isolation and molecular characterization of non-mammalian neural progenitors are largely hampered due to the lack of an appropriate cell surface marker. Taking advantage of the molecular information obtained within the mammalian brain [3.33,34], we have addressed whether characterization of the stem cell marker prominin-1 (CD133) could provide essential information, if not a signature, of germinative (proliferating) neurogenic zones of the non-mammalian vertebrate CNS. Recently, prominin-1 orthologs were isolated from non-mammalian vertebrate model organisms (chick, zebrafish, axolotl and frog) providing unique biological tools for such an investigation [35], [36]. By mapping the distribution of their transcripts, the spatial compartmentalization of prominin-1–positive cells was analyzed in relation to proliferating cells. To further strengthen the comparative character of this study, an anatomical distribution of prominin-1–positive cells within the developing and postnatal murine CNS is provided.

Collectively, this study demonstrates that several features of the distribution of prominin-1–positive cells in the non-mammalian vertebrate CNS are markedly reminiscent to those in mammals. Indeed, their expression highlight neurogenic germinative zones within the brain of all species investigated. Nevertheless, subtle differences are observed as to their appearance at distinct extraventricular (parenchymal) locations. Finally, an up-regulation of prominin-1 is detected upon tail amputation in axolotl suggesting the role of prominin-1–positive cells in compensatory neurogenesis during spinal cord regeneration.

## Materials and Methods

### Ethics Statement

Animal experiments were performed in strict accordance with German Animal Welfare legislation, and all procedures were performed in compliance with the UK Animals (Scientific Procedures) Act 1986, which were approved by the Institutional Animal Welfare Officer (Tierschutzbeauftragter). All efforts were made to minimize the suffering of animals, and those ones for organ removal were sacrificed according to the “Schedule 1” method of humane killing. Max-Planck-Institute of Molecular Cell Biology and Genetics holds necessary licenses (74-9165.40-9-2000-1 and 74-9168.25-9-2001-1) for keeping and breeding of laboratory animals and collecting organs and tissues, both issued by Regierungspräsidium Dresden, Saxony. The Institutional Animal Welfare Officer (Tierschutzbeauftragter) approved all organ collection experiments from wild-type animals, involving no genetically modified organisms, bound to regular reporting under the licenses above.

### Tissue Preparation

#### Mouse

Murine tissue samples were obtained from NMRI strain. The day of vaginal plug was considered as embryonic day 0.5 (E0.5). Timed pregnant mice and postnatal animals at day 0 and 10 (P10) were deeply anesthetized by a single intra-peritoneal bolus injection of Ketamine and Xylazine mixture. Postnatal animals were then trans-cardially perfused with ice-cold 4% paraformaldehyde (PFA) in phosphate buffer pH 7.4. Brains were removed and post-fixed in 4% PFA for 2 h at 4°C. Litters were dissected and embryos at different stages of development were fixed by immersion in 4% PFA overnight at 4°C. After cryoprotection with 30% sucrose-PBS, tissue samples were embedded in OCT compound (Tissue-Tek, Sakura, The Netherlands). Tissues were sectioned on a cryostat (HM560, Microm International GmbH, Walldorf, Germany) at 10 μm and then mounted onto SuperFrost® Plus microscope slides (Menzel-Gläser, Braunschweig, Germany), dried overnight at room temperature, and stored at −20°C until use.

#### Chicken

Fertilized eggs of white Leghorns were purchased from an aviary (Cuxhaven, Germany). Eggs were incubated in a humidified egg chamber at 38°C until embryonic day 10 (E10; HH36 stage according to Hamburger and Hamilton, [37]), E15 (HH41) and E20 (HH45). At the indicated day of incubation whole embryos were harvested. Heads of E10 embryos were fixed by immersion in 4% PFA overnight at 4°C, whereas embryos of more advanced stages were trans-cardially perfused with ice-cold 4% PFA. Brains were dissected from the skull and post-fixed in 4% PFA for 2 h at 4°C. After cryoprotection with 30% sucrose-PBS, tissue samples were embedded in OCT compound (Tissue-Tek), sectioned at 14 μm and mounted.

#### Axolotl

Axolotls (*Ambystoma mexicanum*) were bred at the Animal Facility of the MPI-CBG. They were kept at 18°C in tap water and fed daily with *Artemia nauplia*. Following an overdose of 0.03% Tricaine methanesulfonate (Ethyl 3-aminobenzoate methansulfonate [MS-222]; E-10521; Sigma-Aldrich, St. Louis, MO), brains were dissected from the skull of juvenile axolotl (length of 7.5 cm) and were fixed by immersion in 4% PFA overnight at 4°C. After cryoprotection with 30% sucrose-PBS, tissue samples were embedded in OCT compound (Tissue-Tek). Samples were sectioned at 14 µm and mounted.

For the tail amputation, 3.5-cm long larval axolotls were deeply anesthetized with 0.01% Tricaine methanesulfonate. Three myotomes post-cloacally, the tail was removed under an Olympus SZX12 binocular microscope. The post-cloacal tail segments were fixed by immersion in 4% PFA overnight at 4°C for further analysis. The amputated animals were allowed to regenerate in Holtfreter's solution (www.axolotl.org), and after five days of healing were sacrificed. The regenerated tail fragments were fixed in 4% PFA overnight at 4°C. Materials from normal and regenerated tails were cryoprotected, embedded and sectioned at 14 µm.

#### Zebrafish

Fish (*Danio rerio*) were raised and kept under standard conditions at 27°C [38], [39] at the Animal Facility of the MPI-CBG. Adult, sexually matured 3 month-old fish from the AB genetic background were used [40]. Brains were removed from the skull with fine tip watchmaker's forceps following an overdose of 0.1% Tricaine methansulfonate solution. Tissue samples were fixed by immersion in 4% PFA overnight at 4°C. After cryoprotection with 30% sucrose-PBS, tissue samples were embedded in OCT compound (Tissue-Tek). Samples were sectioned at 14 µm and mounted. To reveal the actively proliferating foci in the brain, fish were incubated in 10 mM 5-bromo-deoxyuridine (BrdU) solution in E3 medium (5 mM NaCl, 0.17 mM KCl, 0.33 mM CaCl_2_, 0.33 mM MgSO_4_ and 10^−5^ % (w/v) Methylene Blue; pH 7.5) for 48 h prior tissue preparation [41]. The BrdU incorporation was detected by immunostaining (see below).

### Non-Radioactive In situ Hybridization

In situ hybridization (ISH) on PFA-fixed cryosections was performed according to a protocol as described [35]. Briefly, serial sections were hybridized with appropriate species-specific digoxygenin (DIG) labeled cRNA probes (see below) at a concentration of 0.5 ng/µl for 16 h at 70°C. Stringent washes were performed at 70°C. The sections were then incubated with anti-DIG antibody (1∶4,000; Roche Molecular Biochemicals, Mannheim, Germany) for 16 h at 4°C. After several washing steps the reaction was visualized using NBT-BCIP substrate (Roche Molecular Biochemicals). After stopping the reaction by several washes in PBS, sections were counterstained with 4,6-diamidino-2-phenylindole (DAPI; 1 µg/ml; Molecular Probes). Sections were rinsed with PBS followed by a quick rinse in dH_2_O. Then the slides were either mounted with Kaiser's Glycerol-Gelatin (Merck, Darmstadt, Germany) or processed for immunohistochemistry (IHC; see below). From every specimen at a given time of development, we routinely processed more than four sections per marker. For each developmental stage, more than three animals were sacrificed.

### Probes for In situ Hybridization

Antisense complementary ribonucleic acid (cRNA) probes were generated using DIG labeling mix (Roche Molecular Biochemicals). To synthesize murine prominin-1 (Accession Number AF026269) and prominin-2 (Accession Number AF269062) cRNA probes a 2.1 kb (nt 198–2264) and a 1.9 kb (nt 346–2258) cDNA fragment was used, respectively. For the cRNA probes of non-mammalian vertebrates, cDNA inserts containing the full coding sequence of *Dr* prominin-1a (HQ386793), *Am* prominin-1 (DQ285041) and *Gg* prominin-1 (HQ386789), except for *Dr* prominin-1b (nt 234–1,599; AF373869) were used. The average homology of the *Dr* prominin-1 co-paralogs along the stretch used for ISH is ≈62%. Combined with the stringent hybridization and post-hybridization conditions, these preclude any cross-hybridization. *Dr* musashi-1 probe was previously described [35].

### Combined In situ Hybridization and Immunohistochemistry

Combined ISH/IHC was performed as previously described [42]. After completion of the ISH procedures, tissue samples were incubated with either mouse anti- proliferating cell nuclear antigen (PCNA) monoclonal antibody (mAb) (1∶500; Clone PC10, code M0879; DAKO, Glostrup, Denmark) or rabbit antiserum directed against Olig-2 (1∶1,000; Cat. number AB9610, Chemicon, Millipore, Billerica, MA). For samples derived from chicks, the primary antibody was detected using Alexa Fluor^®^488-conjugated goat anti-mouse secondary antibody (1∶750; Molecular Probes, Invitrogen), whereas those derived from axolotl and murine tissues biotin-conjugated horse anti-mouse (for PCNA) or goat anti-rabbit (for Olig-2) secondary antibodies (1∶500; Vector Laboratories, Burlingame, CA, USA) were used. These biotinylated antibodies were detected using the avidin-biotin-peroxidase complex (Vectastain^®^ Elite ABC kit; Vector Laboratories, Burlingame, CA) and 3,3′ -diaminobenzidine (DAB; 2 µg/ml; Fluka, Darmstadt, Germany) chromogen according to the manufacturer's instruction. The slides were mounted with Mowiol 4–88 (Calbiochem^®^, Merck, Darmstadt, Germany). For BrdU detection, sections were treated twice with 2 M HCl for 10 min at 37°C before the incubation with the rat anti-BrdU mAb (1∶200; clone BU1/75 (ICR1), Abcam, Cambridge, UK). Haematoxylin-eosin staining was performed according to standard procedures using Mayer's haematoxylin and eosin.

### Microscopy Analyses

Stained serial sections were observed using an Olympus BX61 microscope with the IPLAB software. The composite images were prepared from the digital data files using Adobe® Photoshop and Illustrator software (San Jose, CA).

## Results

The spatial distribution of prominin-1 and its relation to germinative zones were investigated in the developing and postnatal CNS of distinct vertebrate classes by non-radioactive ISH. Detection of prominin-1 transcripts allows the direct visualization of cells expressing this molecule independently of distant projections of plasma membrane protrusions, where the protein product is concentrated [43]. Thus, evaluating prominin-1 expression based solely on the protein that is often localized far away from the cell soma might lead to false negative scoring of given cell types.

### Prominin-1–Expressing Cells in the Developing and Postnatal Murine Brain

Until now detection of prominin-1–positive cells in the mammalian brain is only scarcely documented and the available information is scattered and fragmented [15], [33], [44], [45]. Consequently, distribution of prominin-1–positive cells within the pre- and postnatal murine CNS was investigated to draw a comparative picture on expression of prominin-1 across species.

**Figure 1:**
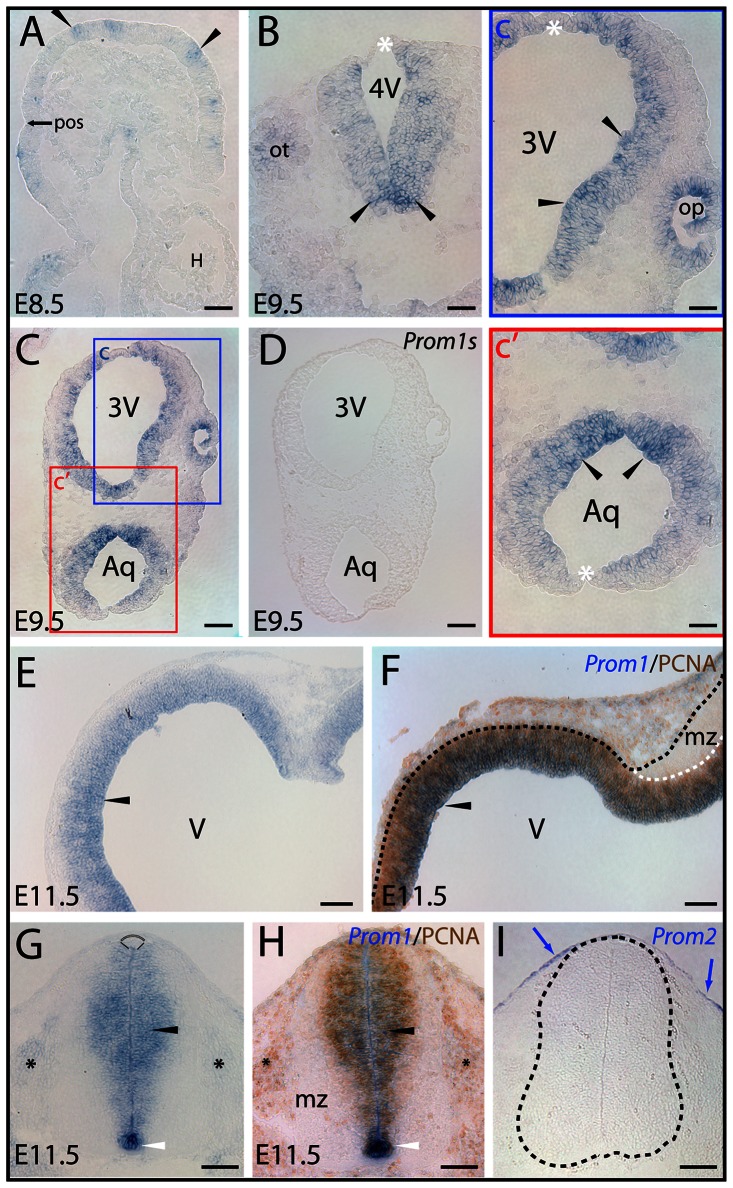
Distribution of murine prominin-1–positive cells in neural plate and developing neural tube. (A–I) Cryosections of mouse embryos at early stages of development as indicated (E8.5, E9.5 and E11.5) were processed for ISH using either antisense (A–C, E, G) or sense (D; *Prom1s*) DIG-labeled prominin-1 probe alone or combined with immuno-detection of PCNA (F, H; *Prom1*/PCNA). Prominin-2 was detected with an antisense probe (I; *Prom2*). The boxed areas in C are shown at higher magnification in panels c and c'. Black arrowheads indicate prominin-1–positive neuroepithelial cells (blue) found as clusters in the anterior neural plate before neurulation (A) or thereafter along the neural tube within the hindbrain (B) and mesencephalic vesicle (C, c'), the prospective diencephalic part of the prosencephalon (C, c), and at later stage in the telencephalic vesicle (E), the mesencephalic-hindbrain junction (F) and ventricular zone of the spinal neural tube (G) where overlapping signals with PCNA (brown) are observed (F, H). Prominin-1–positive cells are also detected in otic (B; ot) and optic (c; op) vesicles, and prospective dorsal root ganglia (G, H; black asterisks). Note the intensity of prominin-1 expression within the neural tube is decreasing from the ventral midline floor (B, C, G, H; arrowheads) towards the dorsal roof plate (B, C, G; white asterisks, arc). The mantle zone (mz) is negative for prominin-1 and PCNA (F, H). Black and white dashed lines delineate the neural tube and its mz (F, I). Blue arrows indicate prominin-2–positive cells at the surface ectoderm whereas neural tube is negative (I). Orientation: A, parasagittal section – anterior to the right; E, coronal section – dorsal at the top; F, horizontal section – caudal to the right. 3V, prospective 3^rd^ ventricle; 4V, prospective 4^th^ ventricle; Aq, prospective aqueductus cerebri; H, heart tube; pos, pre-otic sulcus; V, prospective lateral ventricle. Scale bars, A, B, c, c', 50 µm; C–I, 100 µm.

Prominin-1–expressing cells were detected in a salt-and-pepper like pattern in the prospective forebrain of 8.5 day-old embryonic (E8.5) animals, i.e. at the neural plate stage before formation of the neural tube (Fig. 1A, arrowheads). After the closure of neural tube (E9.5), prominin-1–expressing cells became more abundant as shown in the rhombencephalic, mesencephalic and prosencephalic vesicles of the neuraxis (Fig. 1B, C, insets c an c', arrowheads). However, their distribution along the dorsoventral axis was not homogenous. Cells located closer to ventral co-ordinates, i.e. those derived from the basal plate, were more intensely stained than the ones located closer to, or within, the dorsal midline (Fig. 1B, C). The ectoderm-derived otic- and the evaginating neuroepithelial optic vesicles contained prominin-1–expressing cells as well (Fig. 1B, ot; Fig. 1C, inset c, op, respectively).

In E11.5 embryos, prominin-1 transcripts were broadly expressed in the ventricular zone (Fig. 1E, black arrowhead) given the pseudostratified arrangement of neuroepithelial progenitors. By contrast, prominin-1 protein is confined to a narrow stripe lining the luminal surface of brain vesicles [33]. Prominin-1–positive cells overlapped with proliferating progenitors as indicated by detection of PCNA (Fig. 1F, black arrowhead). The mantle zone containing postmitotic cells was devoid of both signals (Fig. 1F, mz). In the spinal neural tube, a similar distribution was observed, i.e. prominin-1–positive cells were aligned along the ventricular progenitor zone (Fig. 1G, black arrowhead). However, the relative expression of prominin-1 along the dorsal/ventral axis was inhomogeneous. Thus, the ventral midline floor was particularly enriched of transcripts, the ventricular zone of the dorsal neural tube (alar plate), especially the roof plate (Fig. 1G, arc and demarcating black lines), was faintly stained. Again, a significant overlapping distribution was observed for prominin-1 labeling and PCNA–positive progenitors (Fig. 1H, black arrowhead). No staining was observed in the mantle zone containing postmitotic neuronal cells (Fig. 1G), and only a weak expression was detected in the neural crest-derived developing dorsal root ganglia (Fig. 1G, H, black asterisks). In contrast to prominin-1, prominin-2–expressing cells were confined to the surface ectoderm (Fig. 1I, blue arrow). In E14 embryos, prominin-1–positive cells were broadly distributed in the ventricular zone of the telencephalic compartment ( Fig. S1A–C). They were detected in the medial, dorsal and lateral *pallium* (Fig. S1A–C, black arrowheads) as well as in ganglionic eminences (Fig. S1B, blue arrowhead). The mantle zone was negative (Fig. S1A, C, hollow arrowhead). Interestingly, the epithelial anlagen of the developing choroid plexus of lateral ventricles were also devoid of prominin-1 (Fig. S1B, cp). Along the diencephalic ventricle (i.e. 3^rd^ ventricle) the staining intensity appeared to be weaker and the distribution of prominin-1–positive cells was inhomogeneous (data not shown).

**Figure 2:**
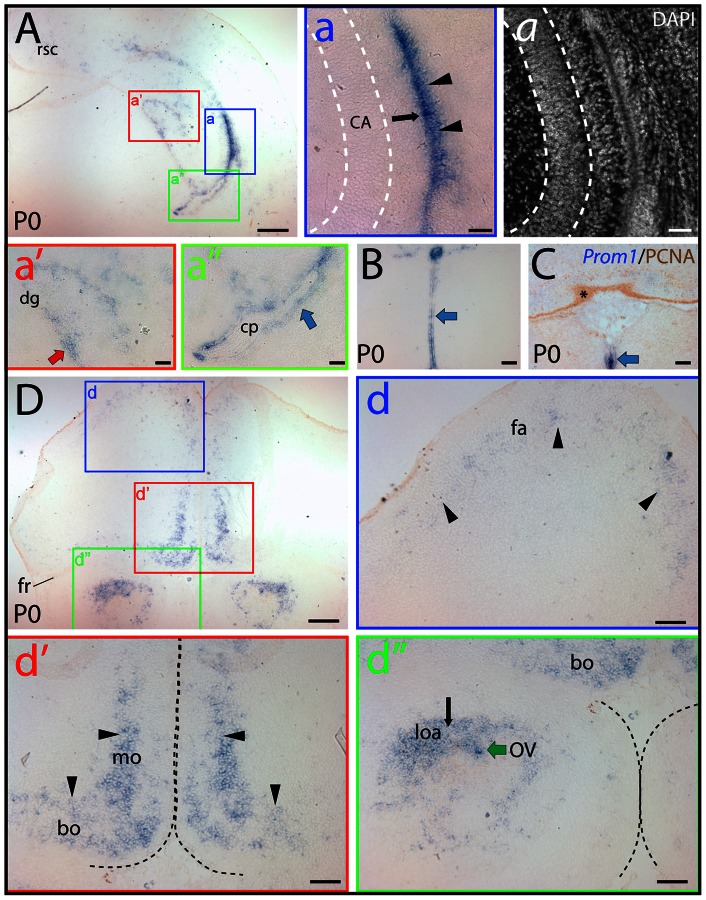
Distribution of prominin-1–positive cells in the brain of newborn mouse. (A–D) Cryosections of brains from postnatal day 0 (P0) animals were processed for ISH using an antisense DIG-labeled prominin-1 probe (A, B, D) alone or combined with immuno-detection of PCNA (C; *Prom1/*PCNA). The nuclear architecture was revealed using DAPI (*a*). The boxed areas in A and D are shown at higher magnification in the respective panels (a–a”, d–d”). (A, B, C) Cross-sections of telencephalic hemisphere at level of the commissura posterior (A), diencephalon (B) and hindbrain (C) reveal the restricted expression pattern of prominin-1–positive cells (blue) at the hippocampal and neopallial germinative sides of the lateral ventricle (a; black arrow and arrowheads, respectively), in the developing dentate gyrus (a'; dg, red arrow), the ependymal layers (blue arrows) lining the lateral (a”) and 3^rd^ ventricle (B) as well as the midline floor of the 4^th^ ventricle (C). Choroid plexus epithelium (a”; cp) and the germinative layer of the cerebellum marked by PCNA (C; brown, asterisk) are negative. (D) Rostral cross-section of the telencephalon near to the frontal pole reveals that prominin-1–positive cells form an irregular stripe extending from the dorso-lateral through the medial to the basal (orbital) sides, where the labeling intensity is increasing toward the basal domain (compare panels d and d'; arrowheads). Black and green arrows indicate prominin-1–positive cells in the lateral olfactory area (loa) and in the ependymal wall of the olfactory ventricle (OV), respectively **(**d”). White dashed lines delineate the pyramidal layer of the hippocampus (a) whereas the black ones separate the two hemispheres (d') and olfactory bulbs (d”). Bo, basal orbito-frontal cortex; CA, Cornu Ammonis; fa, frontal association cortex; fr, Fissura rhinalis; mo, medial orbito-frontal cortex; rsc, retrosplenial cortex. Scale bars, A, D, 250 µm; a, *a*, 50 µm; a', a”, B, C, d–d”, 100 µm.

In newborn mice (i.e. P0), the general abundance of prominin-1–positive cells was decreased as the embryonic ventricular zone neurogenesis consumes most of the progenitors by the early postnatal life indicating a potential link between prominin-1 expression and proliferation of neural progenitors. Significant neurogenesis in the postnatal brain is confined to the ontogenetically delayed developing parts, i.e. hippocampus formation and cerebellum [46]. Indeed, a marked expression of prominin-1 was observed in the hippocampal and neocortical side of the lateral ventricle wall (Fig. 2A, a, black arrow and arrowheads, respectively). Therein the labeling was stronger in the lateral wall of the ventricle, where the germinative cells were reported to be more numerous, than on the opposite hippocampal side. Prominin-1–positive cells were also detected in the forming dentate gyrus (Fig. 2A, a', red arrow). Expression of prominin-1 was otherwise rather sporadic in the remaining ependymal cells lining the ventricle system as exemplified by the lateral ventricle (Fig. 2A, a”, blue arrow), 3^rd^ ventricle (Fig. 2B; blue arrowhead) and the *fossa rhomboidea* of the 4^th^ ventricle (Fig. 2C; blue arrowhead). Outside the ventricle system (i.e. in the brain parenchyma), a weak prominin-1 expression was detected in the frontal association cortex (Fig. 2D, d, fa, arrowheads), and a modest one in the fronto-basal and medial orbital cortex (Fig. 2D, d', bo and mo, respectively). Prominin-1 transcripts were also detected in the lateral olfactory area and, as part of the ventricle system, in the ependymal lining of the shrunken olfactory ventricle (Fig. 2D, d”, loa and OV, respectively). Interestingly, the proliferating PCNA–positive external granule cells in the developing cerebellum were negative for prominin-1 (Fig. 2C, asterisk).

**Figure 3:**
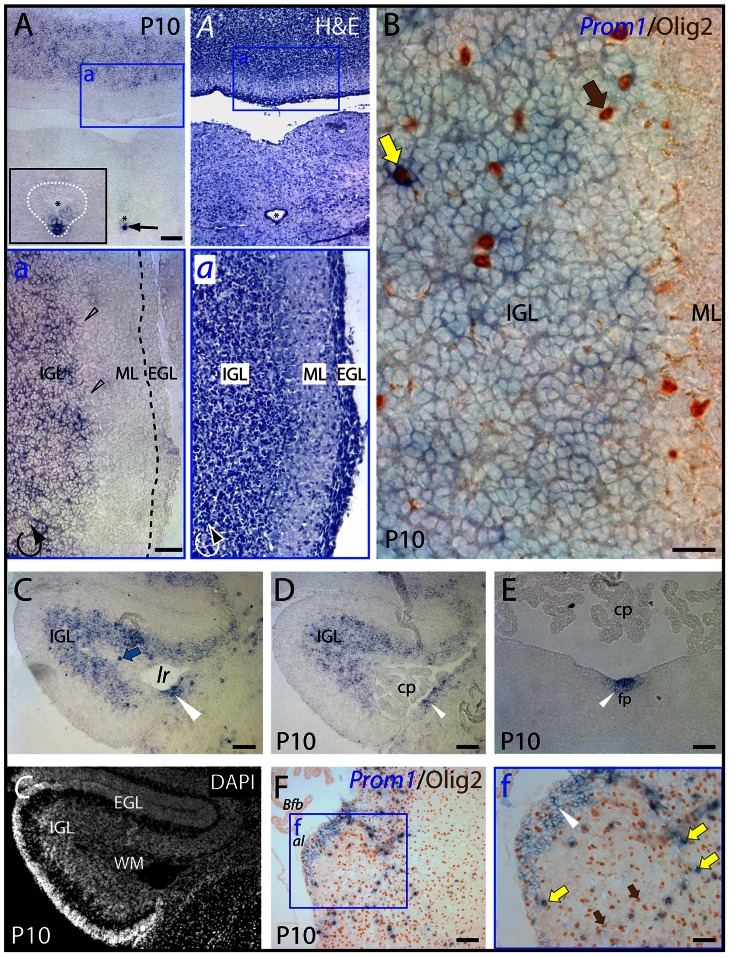
Distribution of murine prominin-1–positive cells in the cerebellum and hindbrain. (A–F) Cryosections of brains from postnatal day 10 (P10) animals were processed for ISH using an antisense DIG-labeled prominin-1 probe (A, C–E) alone or combined with immuno-detection of Olig2 (B, F; *Prom1/*Olig2). The cytoarchitecture was revealed using haematoxylin-eosin (H&E) straining (*A*, *a*) or DAPI (*C*). The boxed areas in A, *A* and F are shown at higher magnification in the respective panels a, *a* and f. (A, B) Cross-sections through the caudal medulla oblongata and cerebellum show prominin-1–positive cells (blue) in the cerebellar inner granule layer (IGL) and their absence from the molecular (ML) and external granule layers (EGL; both delineated by black dashed line in sub-panel a). Note a strong prominin-1 expression (black arrow) in ependymal cells (white dashed line) lining the ventral midline of the central canal (asterisk, see inset in panel A). (**C**–**F**) Cross-sections of the rostral medulla oblongata through the lateral recess (*lr*) (C, D) and midline floor of the fossa rhomboidea (E) at the level of the cerebellar flocculus (C–E) or at the cochlear area/lateral aperture (F) reveal prominin-1–positive cells in both subependymal and ependymal locations (white arrowheads) underlying the lateral recess or in the floor plate (fp). Prominin-1 is also detected in the cerebellar white matter (C, *C*; WM, blue arrow), and scattered prominin-1–positive cells expressing Olig2 (brown; yellow arrows) are observed within the cerebellum (B) and hindbrain parenchyma (f). Purkinje-cells (black hollow arrowheads), choroid plexus (*cp*) and its Bochdalek's flower basket (*Bfb*) are negative. Olig2–positive/prominin-1–negative cells are indicated with brown arrows. al, *apertura lateralis* Luschkae. Scale bars, A, a, C–F, 100 µm; B, 25 µm; f, 50 µm.

In ten-day-old animals, however, the cerebellum displayed an intense labeling where prominin-1–positive cells were confined to the internal granule layer (Fig. 3A–D, IGL). The cells within the germinative external granule layer (Fig. 3A–D, EGL) and molecular layer (Fig. 3A, B, ML) as well as Purkinje cells (Fig. 3a, black hollow arrowheads) were devoid of prominin-1. The choroid plexus within the fourth ventricle and outside of it (i.e. Bochdalek's flower basket bulging out at the lateral aperture) was negative (Fig. 3C–F, cp and *Bfb*, respectively). In the hindbrain, a strong expression of prominin-1 was observed in ependymal cells along the ventral midline of the central canal (Fig. 3A, arrow, see inset) and *fossa rhomboidea* (Fig. 3E; white arrowhead) reminiscent of earlier stages (see above). Likewise, a local enrichment of prominin-1–positive cells was observed on the dorsolateral surface of the hindbrain corresponding very likely to the neurogenic caudal rhombic lip compartment, which is known to be responsible for the generation of cochlear nuclei (Fig. 3C, D, white arrowhead). Finally, it is interesting to note that a scattered minute population of prominin-1–positive cells co-expressing the bHLH-transcription factor Olig2, a marker of the oligodendroglia lineage, was observed in the internal granule layer of the cerebellum (Fig. 3B, yellow arrow) and in the hindbrain parenchyma (Fig. 3F, yellow arrows). Moreover, prominin-1 expressing cells were detected in the cerebellar white matter (Fig. 3C, WM, blue arrow) in agreement with the expression of prominin-1 protein in certain subpopulation of glial cells as demonstrated by its detection in myelin sheaths of adult mice [45].

### Distribution of Prominin-1–Positive Cells in the CNS of Non-Mammalian Vertebrates

#### Chicken

**Figure 4:**
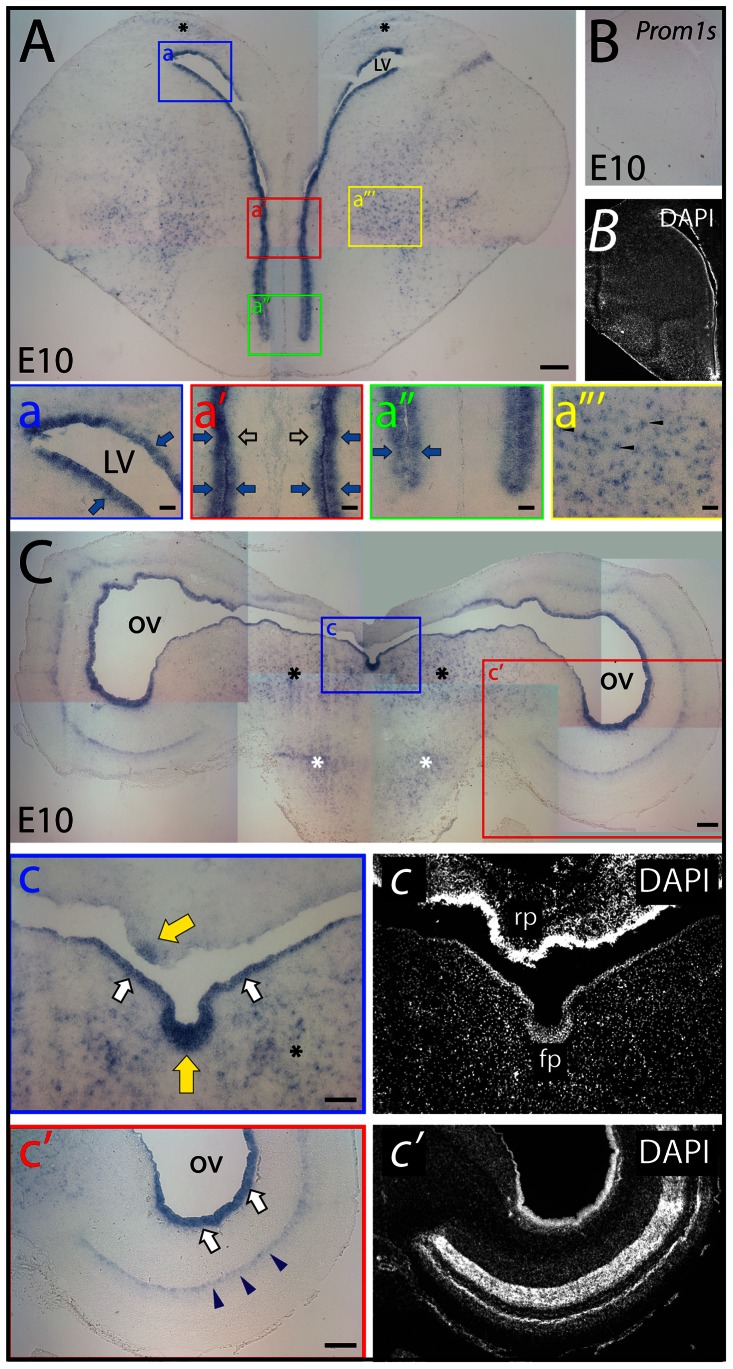
Distribution of prominin-1–positive cells in telencephalon and mesencephalon of embryonic chick. (A–C) Cryosections of brains from chick embryos at day 10 (E10) were processed for ISH using either an antisense (A, C) or sense (B; *Prom1s*) DIG-labeled prominin-1 probe. The nuclear architecture was revealed using DAPI (*B, C, C*'). The boxed areas in A and C are shown at higher magnification in the respective panels a–a'” and c–c'. Coronal cross-sections from the rostral telencephalon (A) reveal prominin-1–positive cells (blue) all along the ventricular zone (A, a–a”; blue arrows). Note the reduced level of prominin-1 expression at the transition of the ventral germinative zone and medial septal ventricular wall (A, a'; black hollow arrows). Cross-section profiles of the mesencephalon (C) reveal prominin-1–positive cells along the ventricular zone lining the tegmentum (c) and tectum (c') (white arrows), being especially enriched in the midline floor (mf, yellow arrow) and less intensive in the roof plate (rp, yellow arrow). A thin sub-layer of cells in developing optic tectum is positive (blue arrowheads). Scattered parenchymal prominin-1–positive cells are detected both in the pallium (A; asterisks) and subpallium (a”'; arrowheads) as well as in the mesencephalic and pontine tegmentum (C, c; black and white asterisks, respectively). LV, lateral ventricle; OV, optic ventricle. Scale bars, A, C, c', 250 µm; a–a'”, 50 µm; c, 100 µm.

In E10 chicken brain, prominin-1–positive cells were found along the ventricular zone of telencephalon and mesencephalon (Fig. 4A, C, respectively). Distribution of transcripts was, however, inhomogeneous. In the telencephalon, a strong prominin-1 expression was observed in the ventral germinative zone; while further along the medial ventricular wall towards the septum its significant reduction was detected (Fig. 4a', black hollow arrows). In the mesencephalon, its expression was particularly strong at the midline floor and weak at the roof plate (Fig. 4c, *c*, mf, rp, yellow arrows). Beside ventricular zone expression, significant populations of prominin-1–positive cells were observed in the brain parenchyma. Thus, they were detected both in the pallium (Fig. 4A, asterisks) and subpallium (Fig. 4a”', arrowheads) of the telencephalon as well as in the mesencephalic and pontine tegmentum (Fig. 4C, black and white asterisks, respectively) reminiscent of distribution of double prominin-1/Olig2–positive cells observed in postnatal mice (see above, [45]). Interestingly, prominin-1–positive cells were also formed a thin sublayer within the developing optic tectum aligned along the innermost aspect of a transient embryonic lamina known to give rise to the “h-i-j” laminae of the *stratum griseum et fibrosum superficiale* (Fig. 4c', blue arrowheads).

**Figure 5:**
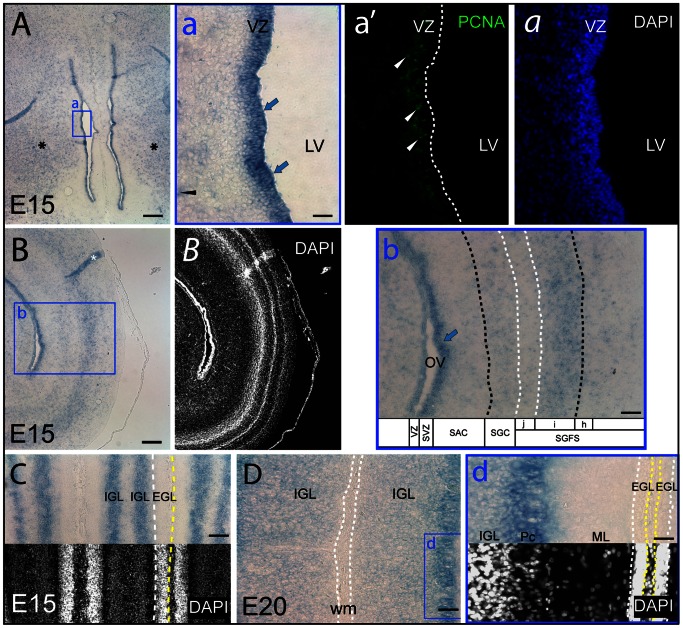
Distribution of prominin-1–positive cells in telencephalon, optic tectum and cerebellum of embryonic chick. (A–D) Cryosections of brains from chick embryos at day 15 (E15) and 20 (E20) were processed for ISH using an antisense DIG-labeled prominin-1 probe alone (A–D) or combined with immuno-detection of PCNA (a'; PCNA). The nuclear architecture was revealed using DAPI (*a*, *B*, C, d). The boxed areas in A, B and D are shown at higher magnification in panels a, b and d, respectively. (A, B) Coronal sections of the rostral telencephalon (A) and a hemisphere of the mesencephalic optic tectum (B) reveal prominin-1–positive cells (blue arrows) along the telencephalic and tectal ventricular zone (VZ), respectively, where proliferating cells are detected (a'; white arrowheads, PCNA). Parenchymal (extraventricular) prominin-1–positive cell populations are found both in the telencephalic pallium and subpallium (A; black asterisks, a; black arrowhead) as well as in all sublayers of the tectum being especially enriched in the stratum griseum centrale (SGC) and lamina i of the stratum griseum et fibrosum superficiale (SGFS) (B, b; optic tectum layers are indicated by dashed lines). (C, D) Sections through cerebellar folia reveal the presence of prominin-1–positive cells in the cerebellar inner granule layer (IGL) and Purkinje cells (Pc) and their absence from the molecular layer (ML) and the subpial external granule layer (EGL, white and yellow dashed lines). Tectal sublayers were identified by the nuclear architecture according to the literature [102], [103] White matter (wm) in the centre of a cerebellar folium is indicated with dashed lines. LV, lateral ventricle; OV, optic ventricle; SAC, stratum album centrale; SVZ, subventricular zone. Scale bars, A, B, 250 µm; a, d, 25 µm; b, C, 100 µm; D, 50 µm.

In older embryos (i.e. E15, E20), the distribution pattern of prominin-1–positive cells was in many respects similar to what has been described for the E10 brain. Accordingly, in the telencephalon, they were not only confined to the germinative PCNA–positive ventricular zone (Fig. 5A, a, a'; blue arrows and white arrowheads, respectively), but also detected in extraventricular regions (Fig. 5A, asterisks). By comparison to early stages, they were strikingly expanded in the brain parenchyma (Fig. 5a, black arrowhead). Likewise, a widespread distribution of prominin-1–positive cells was detected in all sublayers of optic tectum (Fig. 5B). Their highest densities coincided with *stratum griseum centrale* and lamina “i” of *stratum griseum et fibrosum superficiale* (Fig. 5b). In the cerebellum, prominin-1–positive cells were found in the inner granule layer and Purkinje cells (Fig. 5C, D, d; IGL and Pc, respectively) and were absent from the molecular and the germinative external granule layers (Fig. 5C, d; ML, EGL, respectively).

#### Zebrafish

In the continuously growing brain of adult teleosts several proliferative foci could be identified within the ventricular zone and proliferative activity, to a much lesser extent, in the parenchyma is also evident [32], [41], [47]–[50]. To dissect the potential relationship between proliferating zones and distribution of zebrafish prominin-1 genes (referred to as prominin-1a and b) a BrdU pulse labeling was applied to young adult fish followed by non-radioactive ISH. For comparison, expression of musashi-1 (msi1) was examined – a member of an evolutionarily conserved family of RNA-binding proteins – that marks various stem cell populations within proliferative foci [51].

**Figure 6:**
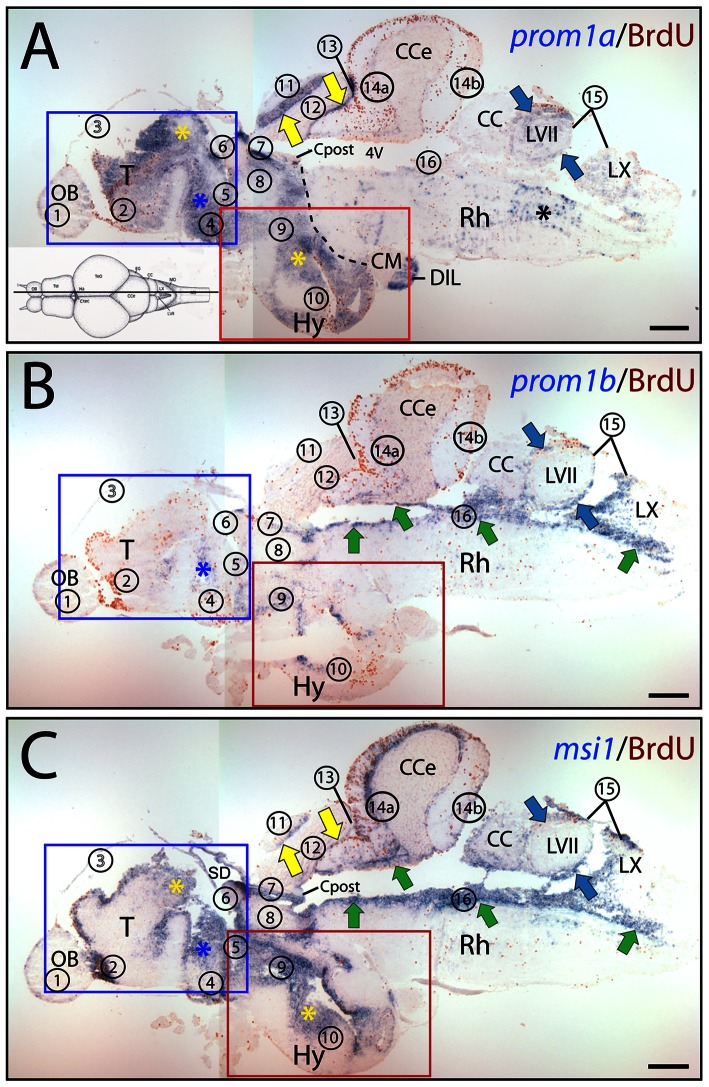
The combined expression of prominin-1a and b mimics the distribution of musashi-1 in adult zebrafish brain. (A–C) Cryosections of 3-month-old adult brain from BrdU-treated zebrafish were processed for ISH using an antisense DIG-labeled probe either against prominin-1a (A; *prom1a*), prominin-1b (B; *prom1*b) or musashi-1 (C; *msi1*). Proliferating cells were observed by immuno-detection of BrdU (brown). Proliferative zones are indicated with Arabic numbers (1 to 16, see below) from rostral to caudal direction along the neuraxis as proposed [41], and position of parasagittal section profiles of the brain is indicated on the cartoon (A) adapted from a standard neuroanatomical atlas of the zebrafish brain by Wulliman and colleagues [104]. The boxed areas in A–C are shown at higher magnification in Figure S2. Black dashed line indicates the border of prosencephalon towards the tegmentum of the brainstem (A). Coloured arrows and asterisks indicate overlapping expression domains of particular genes. Note that major sites of expression of prominin-1a are mainly located in the prosencephalic (rostral) and dorsal mesencephalic (tectal) domain (A), whereas prominin-1b is predominantly found in the rhombencephalic brainstem (caudal) domain (B). Msi1-1–positive cells are confined to all known proliferative zones both in rostral and caudal brain regions (C). CC, crista cerebellaris; CM, corpus mamillare; Cpost, commissura posterior; DIL, diffuse nucleus of the hypothalamic inferior lobe; Rh, rhombencephalon; SD, saccus dorsalis; T, telencephalon; 4V, fourth ventricle. Proliferative zones are: 1, olfactory bulb (OB); 2, ventral telencephalon; 3, dorsal telencephalon; 4, preoptic area; 5, ventral thalamus, 6, habenula; 7, periventricular zone of pretectum; 8, dorsal thalamus; 9, posterior tuberculum; 10, hypothalamus (Hy); 11, tectum opticum; 12, torus longitudinalis; 13, posterior mesencephalic lamina; 14a, molecular layer of valvula and corpus cerebelli (CCe); 14b, lobus caudalis cerebelli; 15, facial and vagal lobes; 16, ventricular zone of the fossa rhomboidea/canalis centralis. Zone 3 is indicated with a hollow number because is not represented on the present section. Black asterisk indicates rhombencephalic brain parenchyma. Scale bars, A–C, 250 µm.

**Figure 7:**
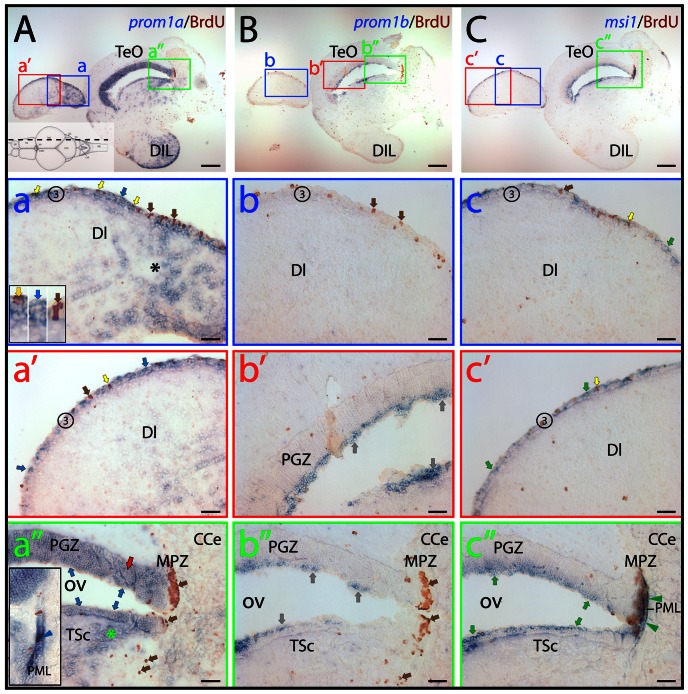
Distribution of zebrafish prominin-1a– and b–positive cells in the dorsal lateral telencephalon and the tectal ventricular zone. (A–C) Cryosections of 3-month-old adult brain from BrdU-treated zebrafish were processed for ISH using an antisense DIG-labeled probe either against prominin-1a (A; *prom1a*), prominin-1b (B; *prom1*b) or musashi-1 (C; *msi1*). Proliferating cells were observed by immuno-detection of BrdU (brown). Position of paramedian longitudinal sections of the brain is indicated on the cartoon (A) adapted from the neuroanatomical atlas by Wulliman and colleagues [104]. The boxed areas in A, B and C are displayed at a higher magnification in panels a–a”, b–b” and c–c”, respectively. (A) Prominin-1a–positive cells are enriched in the diffuse nucleus of the hypothalamic inferior lobe (A, DIL) and in the extraventricular dorsal telencephalic parenchyma (a, black asterisk). They are found within the proliferative zone 3 (3) of the dorsal telencephalic surface (a, a', blue arrows), and are either BrdU–positive or negative (yellow and blue arrows, respectively, see inset in a). Rare proliferating cells are devoid of prominin-1a (a, brown arrow). Prominin-1a–positive cells are also detected in the superficial (blue arrows) and deeper (red arrow and green asterisk) areas of periventricular grey zone (PGZ) and Torus semicircularis (TSc) lining the optic (tectal) ventricle (OV) (a”), respectively. While the marginal proliferating zone of the tectum (MPZ) lacks prominin-1a, a small number of prominin-1a–positive proliferating cells is observed in the posterior mesencephalic lamina (PML) (a”, see inset, blue arrowhead). (B) Prominin-1b–positive cells (grey arrows) are found in the superficial tear of PGZ (b') and in TSc lining the OV (b”), whereas proliferating cells within the zone 3 of the dorsal telencephalic surface (b, brown arrows), the deeper areas of the PGZ (b') and in the MPZ (b”) are negative. (C) Msi-1–positive cells found within the proliferative zone 3 of the dorsal telencephalic surface (c, c') are either BrdU–positive or negative (yellow and green arrows, respectively). Rare proliferating cells are devoid of msi-1 (c, brown arrow). Msi-1 is detected in the superficial tear of PGZ and TSc lining the OV (c”) as well as in proliferating cells of the MPZ and those of the PML (c”, green arrowheads). CCe, corpus cerebelli; DI, dorsal lateral subdivision of the dorsal telencephalic region; TeO, tectum opticum. Scale bars, A–C, 250 µm; a–c”, 50 µm.

**Figure 8:**
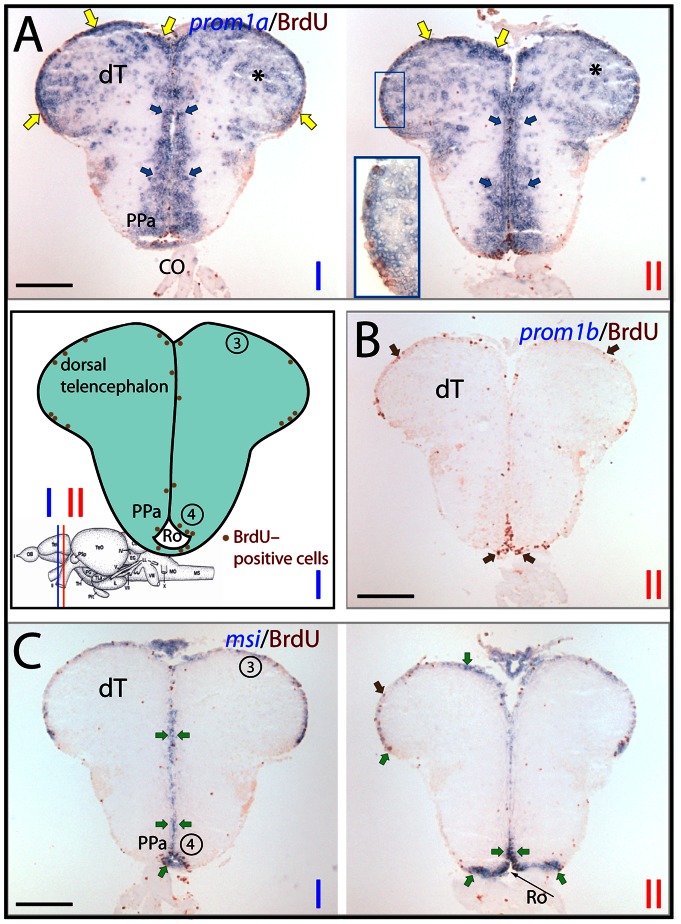
Differential expression of zebrafish prominin-1a– and b–positive cells in the posterior dorsal telencephalon. (A–C) Cryosections of 3-month-old adult brain from BrdU-treated zebrafish were processed for ISH using an antisense DIG-labeled probe either against prominin-1a (A; *prom1a*), prominin-1b (B; *prom1b*) or musashi-1 (C; *msi1*). Proliferating cells were observed by immuno-detection of BrdU (brown). Position of the two coronal cross section profiles (I, II) encompassing the posterior dorsal telencephalic area is indicated on the cartoon adapted from a neuroanatomical atlas by Wulliman and colleagues [104], and the distribution of proliferative events (brown dots) is schematically shown. (A) Prominin-1a–positive cells are distributed along the posterior medial, dorsal and lateral aspects of the dorsal telencephalic surface proliferation zone (yellow arrows, see inset), and in the extraventricular brain parenchyma of the dorsal telencephalon (dT, asterisks) as well in the periventricular preoptic nucleus (PPa, blue arrows). (B) No prominin-1b–positive cells are detected within the dorsal telencephalic and preoptic proliferative zones (brown arrows). (C) Prosencephalic msi1–positive cells are found in a narrow ventricular stripe encompassing the proliferating subdivisions of the telencephalon, and that of the preoptic area and recessus opticus (Ro) of the diencephalic ventricle (green arrows). Proliferative zones 3 and 4 correspond to dorsal telencephalon and preoptic area, respectively. CO, chiasma opticum. Scale bars, A–C, 250 µm.

Taking first the general distribution of the two zebrafish prominin-1 co-orthologs into account, their expression appeared here being uneven along the rostro-caudal axis of the brain, in comparison to msi1. Remarkably, the “sum” of their expression largely recapitulated distribution of msi1 in BrdU-labeled proliferative zones with exception of cerebellum as revealed by the longitudinal overview of mid-sagittal (Figs. 6, S2) and parasagittal sections (Fig. S3). Thus, the prosencephalon (telencephalon+diencephalon) and dorsal mesencephalon were mostly dominated by prominin-1a–positive cells located in proliferative zones therein (Fig. 6A, zones 2/3, 4–10 and 11/12, respectively; see also Figs. S2, S3; proliferative zones are indicated by an Arabic number according to Ref. 41, for details see legend of figure 6), whereas the ventricular zone of the hindbrain was occupied mainly by prominin-1b–positive ones (Fig. 6B, zones 15 and 16, respectively). Nevertheless, both molecules were detectable in proliferating zones of the facial and vagal lobes of the lower brainstem (Fig. 6). Prominin-1b was largely absent from most parts of the telencephalon and mesencephalon (Fig. 6B, zones 2/3, 11/12, respectively; see also Figs. S2, S3). For example, prominin-1b was fully excluded from proliferating zones in the ventral telencephalic area (i.e. subpallium) encompassing a rostral migratory stream-like assembly (Fig. S2B) [32], [41], where prominin-1a and msi1 were strongly expressed (Fig. S2A, C). A similar situation was observed in the dorsal telencephalic area (i.e. pallium) (Fig. S3A–C, zone 3). Indeed, in the posterior pallium, a region considered as the homologue of the archipallial mammalian hippocampus [32], [52], only prominin-1a and msi1, but not prominin-1b, were expressed coincident with the proliferative activity of this area (Fig. 7A–C, zone 3; see also below Fig. 8). It is of note that a modest expression of prominin-1b was solely detected around the diencephalic ventricle system (Fig. 6B; for details see Fig. S2b, b') and in the cerebellum (e.g., valvula cerebelli), reminiscent of msi1 (Figs. 6B, C, zone 14a; S3B, C). In contrast, prominin-1a was absent from the latter structure (Fig. S3A). It is important to point out that neither prominin-1a and -1b transcripts (Fig. 7a and a'; 7b and b', respectively) nor msi1 (Fig. 7c and c') were exclusively confined to BrdU-labeled, dividing cells (Fig. 7a–c'; brown cell nuclei). Indeed, their transcripts were found at numerous occasions in ventricular cells without BrdU incorporation (Fig. 7a–c'; blue reaction).

In addition to the differential expression of prominin-1a and prominin-1b in rostro-caudal direction along the ventricular zone, another major difference between the two prominin-1 genes was the extended expression of prominin-1a observed in subventricular or deeper scattered cells within the brain parenchyma, i.e. far beyond the ventricular system. Hence, prominin-1a–positive cells were found in numerous extraventricular areas including preoptic nucleus (Figs. 8A, PPa; S3A, PO), diffuse nucleus of the hypothalamic inferior lobe and periventricular hypothalamus (Figs. 6A and S3A, DIL; S3A, PVH, respectively). Likewise, they were observed in the extraventricular brain parenchyma of the dorsal telencephalon, especially in its caudal part, (Figs. 7a, 8A, S3A, dT, asterisks) and in the central nucleus of the ventral telencephalic area (Fig. S3A, Vc). Moreover, such cells were also observed in the rhombencephalic parenchyma (Fig. 6A, black asterisk). The broader distribution of prominin-1a–positive cells by comparison to the restricted ventricular localization of prominin-1b– and msi1–positive ones was further exemplified in the tectal ventricular zone (Fig. 7). Although, all three genes were expressed in the periventricular grey zone (PGZ) and torus semicircularis (TSc) around the tectal (optic) ventricle, their expression patterns showed considerable differences. Prominin-1a was found in all differentiated neuronal populations of the PGZ (Fig. 7a”, red arrow) and in cell groups located in the deeper domain of the TSc (Fig. 7a”). In contrast, prominin-1b and msi-1 were confined to the superficial tear of these domains (Fig. 7b”, c”, respectively). Finally, while the marginal proliferating zone of the tectum were negative for prominin-1a and prominin-1b (Fig. 7a”, b”, respectively, MPZ), a small number of prominin-1a–positive cells was nevertheless observed in the posterior mesencephalic lamina just like msi1–positive ones (Fig. 8a”, c”, respectively, PML).

#### Axolotl

A peculiar feature of the pedomorphic brain of ambystomatid salamanders is that the grey matter is not segregated into nuclei or laminae that would be comparable to that of the amniote vertebrates. Consequently, the postmitotic and mature neurons are kept in an embryonic position forming a *stratum griseum* around the circumference of the ventricle, similar to the mantle zone of the embryonic mammalian neural tube. Although, a persistent proliferative activity was described in numerous brain regions of postembryonic axolotl [23], [53], the cellular and molecular composition of the normal growth-associated and homeostatic neurogenic niches are largely unknown.

**Figure 9:**
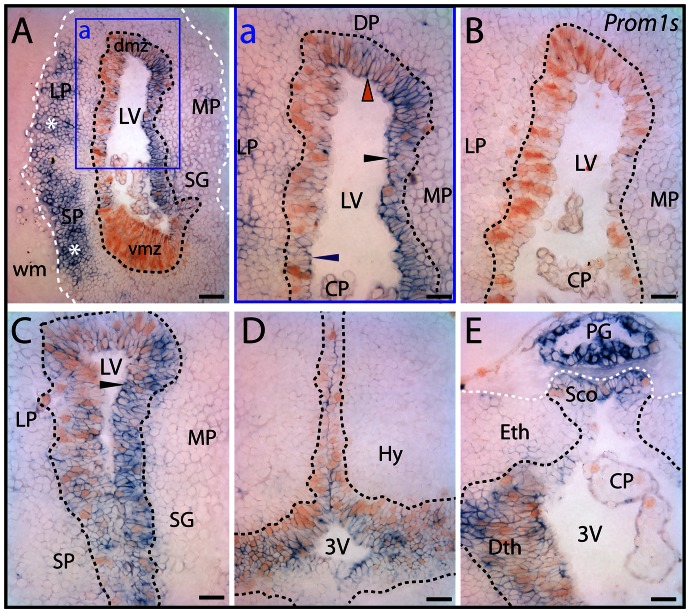
Distribution of prominin-1–positive cells in telencephalon and diencephalon of axolotl. (A–E) Cryosections of the brain from 7.5-cm long juvenile axolotl were processed for ISH using either an antisense (A, C–E) or sense (B; *Prom1s*) DIG-labeled prominin-1 probe combined with immuno-detection of PCNA. The boxed area in A is displayed at a higher magnification in panel a. (A) Coronal section of telencephalic hemispheres rostral to the plane of interventricular foramina reveals an uneven distribution of prominin-1–positive cells (blue) within the proliferating ventricular germinative matrix defined by the PCNA (brown, black dashed lines) and in the surrounding periventricular grey matter. Strong prominin-1 expression is detected in the medial (MP) and dorsal (DP) pallial sector of the ventricular germinative zone (black and red arrowheads, respectively), whereas its expression is modest in the lateral pallial (LP) one (blue arrowhead) and minimal in the ventral matrix zone (vmz). In extraventricular areas, an enrichment of prominin-1 is observed in the LP and subpallial (SP) neuronal assemblies (asterisks) of stratum griseum (SG). Note the paucity of prominin-1 in the choroid plexus (CP) and its absence from white matter (wm, white dashed line). (B) No labeling was observed with sense probe. (C–F) Frontal sections through the caudal telencephalon (C) and those of the ventral (D) and dorsal (E) diencephalon at the level of the subcommissural organ (Sco) reveal prominin-1 expression in proliferating cells (black arrowheads) lining the lateral ventricule (LV) and 3^rd^ ventricle (3V), respectively. A robust prominin-1 expression is detected in the pineal gland (E; PG). The boundary of the grey matter is delimitated with a white dashed line (E). Dmz, dorsal matrix zone; Dth, dorsal thalamus; Hy, hypothalamus; Eth, epithalamus. Scale bars, A, 100 µm; a', C–E, 50 µm.

In the juvenile axolotl brain, prominin-1 was associated with the germinative ventricular zone containing PCNA–positive proliferating progenitor cells (Fig. 9A–E). Interestingly, distribution of neither prominin-1 nor PCNA was homogenous around the ventricular zone. The level of prominin-1 expression varied also with respect to the brain areas (Fig. 9A, C). In the ventricular zone at mid/rostral telencephalic level, for instance, the lateral and dorsal pallium displayed a more intense proliferative activity than the medial one, whereas expression of prominin-1 appeared as being stronger in the latter compartment (Fig. 9a, black arrowhead). Simultaneously, weak and modest prominin-1 expression was found in the lateral and dorsal pallium (Fig. 9a, blue and red arrowhead, respectively). Surprisingly, its expression was almost undetectable in the highly proliferating ventral (subpallial) matrix zone at mid telencephalic level (Fig. 9A, vmz), but became evident towards the more rostral co-ordinates (data not shown). Similarly, intensity of prominin-1 reactivity was significantly increased in the lateral pallium found at the level of the caudal telencephalon (Fig. 9C, for comparison see panel a). Around the ventricular zone of the caudal telencephalon the proliferating events were markedly overlapping with prominin-1 expression. Therein, almost all PCNA–expressing progenitor cells were positive for prominin-1 (Fig. 9C). Interestingly, a significant pool of prominin-1–positive cells were observed in non-proliferating extraventricular areas such as the lateral and subpallial neuronal assemblies of *stratum griseum* (Fig. 9A, asterisks). Double prominin-1/PCNA–positive cells were also detected in ontogenetically more caudal parts of the prosencephalon. Therein, the double-labeled cells were mainly confined to the ventricular zone of dorsal and ventral diencephalic (hypothalamic) regions nearby the third ventricle (Fig. 9D and E, respectively). It is of note that the epithalamic pineal gland and subcommissural organ displayed a strong expression of prominin-1 (Fig. 9E).

Taken together, these data showing expression of PCNA in subpopulations of prominin-1 positive cells within the ventricular zone suggest that prominin-1 might be associated with constitutive neurogenesis. However, its expression in cell populations that are not connected directly to constitutively active proliferation is indicative of further, non-neurogenic, neural function(s) like in all the vertebrate species investigated.

### Expression of Prominin-1 in Response to Injury

Previous reports suggested that phenotypic characteristics of activated ependymal cells during spinal cord regeneration of injured axolotl are strongly reminiscent of the progenitors found in the developing mammalian CNS [reviewed in 22]. Given that, expression of prominin-1 was evaluated in the regenerating spinal neural tube of axolotl following tail amputation.

**Figure 10:**
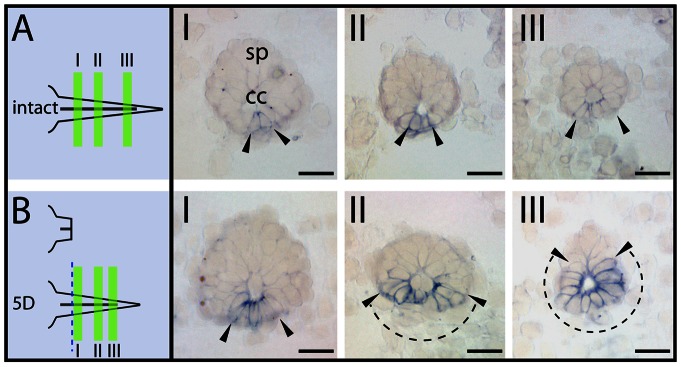
Up-regulation of prominin-1 in regenerating spinal neural tube of axolotl. (A, B) Cryosections of intact tail (A, intact) from 3.5-cm long larval axolotl and those of five-day post-amputation tail regenerate (B, 5D) were processed for ISH using an antisense DIG-labeled prominin-1 probe. The plane of amputation and the position section planes (I–III) along the rostro-caudal axis of the spinal neural tube (sp) are schematically displayed. Note that in the regenerating neural tube (B) an expansion of prominin-1 expression (blue, arrowheads) towards lateral and dorsal co-ordinates is observed in the caudal direction (II-III; dashed lines) in comparison to intact samples (A). cc, central canal. Scale bars, A, B, 50 µm.

In the non-injured spinal neural tube of larval axolotl only a limited number of cells located along the ventral midline, corresponding to the floor plate of the neural tube, expressed at a very low level prominin-1 (Fig. 10A, arrowheads and dashed line delimit the expression domain). In response to amputation, as exemplified following a five-day regeneration, prominin-1 expression was dramatically increased, and remarkably, the positive–area was expanded towards lateral and dorsal co-ordinates of the regenerated spinal neural tube (Fig. 10B, dash line between arrowheads). Thus, these data demonstrate that the general property of progenitors to express evolutionarily conserved neurogenic markers (e.g., prominin-1) is preserved irrespective of the fine details of their morphology.

## Discussion

By exploring the cellular distribution of prominin-1 as a unique cell surface marker of stem and progenitor cells we provide a comprehensive study about germinative zones of the CNS among various mammalian and non-mammalian vertebrates. Three novel findings are reported here. First, we described a conserved prominin-1 expression in proliferative ventricular neurogenic cells, irrespective of the species. Second, distinct prominin-1–positive cell populations were unexpectedly found in various neural extraventricular (i.e. parenchymal) locations in all animal models investigated. Third, prominin-1–positive cells were up- regulated during the epimorphic regeneration of the axolotl spinal cord indicating their potential role in provoked neurogenesis.

### Prominin-1–Positive Cells in the Mammalian Brain

Since its discovery fifteen years ago, prominin-1 as a novel cell surface marker of murine embryonic neuroepithelial cells [33.54], and the biological tools detecting this molecule contributed tremendously to the prospective identification and isolation of cells with neurogenic properties both from the embryonic and postnatal nervous system as well as embryonic stem cell-derived neural progenitor populations [15], [33], [44], [55]–[62]. Thus a thorough documentation of prominin-1 expression becomes a crucial matter not only for understanding biological details of brain development, but also to establish potential stem cell-based treatments for neurodegenerative diseases.

During the early stages of the mammalian brain development, as revealed in the present study, the first sign of expression of murine prominin-1 occurs before the neurulation is completed. Interestingly, distribution of prominin-1–positive cells is irregular at the neural plate stage. Such a patchy expression pattern is evocative of a lateral inhibition mechanism that characterizes certain steps of neurogenesis in anamniote vertebrates [63] and fruit flies [64]. Upon closure of the neural tube, murine prominin-1–positive cells become more abundant along the ventricular progenitor zone and express markers of proliferation. Expression of prominin-1 correlates thus with germinative zones irrespective of the morphological and phenotypic transitions (from early neuroepithelial to later radial glial) observed among neural progenitors during development [3], [45]. These data are also consistent with the initial immunodetection of prominin-1 protein at the ventricular-luminal side of progenitors/radial glial cells in midgestational murine neural tube and in E12 telencephalic vesicles as well as in the developing human brain [15], [33], [58], [65].

By the time of birth, a drastic regression of prominin-1 expression was observed coincident with the consumption of germinative “matrix” (i.e. proliferating progenitors). Besides the radial glia-derived ependyma, prominin-1–positive cells were detected also in late developing structures exhibiting an active postnatal neurogenesis such as the cortical hem-derived dentate gyrus or the olfactory bulb. It is interesting to note, that an increased shedding of prominin-1 containing membrane particles was recently described in the cerebrospinal fluid of patients with temporal lobe epilepsy [66] reflecting an enhanced activity of neural stem cells/hippocampal progenitor cell proliferation associated with several CNS diseases including chronic partial epilepsy [67].

Besides association of prominin-1 with neuronal progenitors of distinct phases of neurogenesis, the present study points out a further one with cell populations of various extraventricular locations in the postnatal brain that might partly belong to glial cell lineages as well as neuronal cells. By the second postnatal week, prominin-1 expression was up regulated and prominin-1–positive cells were often seen as a subpopulation of Olig-2 expressing glial cells (this study; [45]). Whether these cells represent a quiescent pool of oligodendrocyte progenitors derived from the ventricular zone or they are identical to multipotent neural progenitors as reported in sub-cortical white matter of the human brain is not evidenced as yet [68], [69]. Further significant extraventricular populations of prominin-1–positive cells were observed in the inner granule cell layer of the cerebellum. Yet, the germinative external granule layer generating the inner granule cells appeared not to express prominin-1 in agreement with an earlier study performed on P7 cerebella [44]. Interestingly, however, existence of multipotent stem cells in the postnatal cerebellar parenchyma that could be prospectively isolated based on expression of prominin-1 and lack of markers associated with neuronal and glial lineages was formerly described [44]. These purified parenchymal progenitor cells could form self-renewing neurospheres and differentiate into astrocytes, oligodendrocytes and non-granule neurons.

The observed association of prominin-1 with extraventricular cells is very suggestive of additional, non-neurogenic, neural function of this gene (see below). Expression of prominin-1 in terminally differentiated neuroepithelium-derived retinal cells or its detection in myelin sheets of oligodendrocytes further supports this notion [35], [45], [70]. The neuroepithelium-derived cuboidal choroid plexus epithelium, however, lacks prominin-1 consistent with a recent case report showing that choroid plexus carcinoma is negative for prominin-1 [72]. Expression of putative stem- and progenitor markers is often context dependent. Interestingly, other established markers of neural stem/progenitor cells such as Sox2 and msi1 behave similarly prominin-1 inasmuch as they could also be detected in retinal neurons [35], [71]. Therefore a careful evaluation and a combinatorial use of markers either in positive or negative selection paradigms are advisable in identifying stem- and progenitor cells.

In adult mammals, prominin-1 was described being expressed at the apical, luminal side of the ependymal layer both in the murine and human brain [33], [73]. It is generally thought that ependymal cells lining the ventricular zone of the adult telencephalon become non-proliferating [74] while the subventricular zone remains mitotically active and represents a certain reservoir of stem cells throughout the lifespan of the individual [1], [75], [76]. Recent studies brought however significant controversies in terms of the identity of prominin-1–positive stem cells therein. On one hand, for instance, a subpopulation of astrocytes, i.e. type B1 cells, within the subventricular zone was shown to express prominin-1 in a regulated fashion at the liquor contacting apical-ventricular endings [77]. On the other hand, prominin-1–positive ependymal cells were described as an additional, and perhaps more quiescent, stem cell population in the mammalian forebrain, although not all of them expressed this marker [15]. Irrespective of these controversies, the adult liquor contacting subventricular neural stem cells are neither ependymal nor astrocytic per se, but share features of both cell types, which may at least in part explain most of the seemingly contradictory data described. Thus, displaced from the ventricular zone by the epithelial ependymal cells the stem cells might have retained their fundamental embryonic/fetal neuroepithelial (morphologic and molecular) properties [reviewed in 78]. In contrast, the epithelial ependymal cells, although can be stimulated to proliferate and act as neural progenitors, do not retain a self-renewing ability [16].

### Neural Prominin-1–Positive Cells Across Species

In the developing mammalian neural tube both early (neuroepithelial) and late neural progenitors (i.e. radial glia) have an elongated, radial morphology. The apical ventricular surface of neuroepithelial cells is enriched in prominin-1 [33]. Analysis of putative stem cells and proliferating progenitors of constitutive adult neurogenic foci in non-mammalian vertebrates revealed that they share some key phenotypic and morphologic features with mammalian neural progenitors especially with those of fetal stages displaying a radial glial morphology [27], [28]. This notion is also supported by the present study describing localization of prominin-1 in actively proliferating cells of CNS germinative zones from both amniote- and anamniote non-mammalian vertebrate species. Prominin-1 might thus represent a molecular signature common to all proliferative as well as latent/quiescent neurogenic stem cells being conserved during evolution.

The CNS of adult non-mammalian vertebrates is characterized by multiple neurogenic foci found essentially along the whole extent of the cerebral ventricular zone [23,53, reviewed in 24–26]. Interestingly, in adult non-mammalian vertebrates an epithelial ependyma and a germinative compartment are not segregated [24] as observed for the embryonic mammalian nervous system. Instead, the neurogenic radial glial cells line directly the ventricular surface [22], [24], [27], [29]. Despite that, these cells are not uniformly active but rather heterogeneous given that a significant population of them is found in a quiescent, non-proliferative state depending on an actual position along the neuraxis [28]. Conversely, the frequency of proliferating cells is uneven along the ventricular surface [28], [32], [47]. Beside the significant overlap of prominin-1 expression with cell proliferation (i.e. PCNA or BrdU-positive) within the intact brain in all species investigated, the present study revealed that expression of stem and progenitor cell markers is not solely restricted to actively proliferating germinative cells. Thus, msi1, an evolutionarily conserved marker of stem cells could also be expressed in quiescent cells lining the ventricular lumen, as demonstrated here for the zebrafish, as well as prominin-1 in all non-mammalian species investigated. It is interesting to note, however, that even these quiescent radial ependymoglial cells within the ventricular surface can be stimulated to re-enter the cell cycle and act as mutipotential progenitors as exemplified by a spinal cord lesion in adult zebrafish [30] or in a toxin-induced lesion/regeneration model of midbrain dopaminergic neurons in metamorphosed newt [31]. The heterogeneity of cells along the ventricle is further complicated by the regionally distinct expression of the two zebrafish prominin-1 co-orthologs [35], [79]. Distribution of the two molecules (prominin-1a and prominin-1b) along the rostro-caudal axis of the ventricle system of the zebrafish brain displays a fairly complementary pattern with only a low-degree of overlapping. Nevertheless, the “sum” of their expression largely recapitulates the general expression of msi1. Indirectly, this is also suggestive for a certain sub-functionalization of the two prominin-1 co-orthologs (see below).

### Prominin-1–Positive Cells and Neural Regeneration

Understanding the molecular and cellular mechanisms underlying the neural regeneration in non-mammalian vertebrates (e.g. axolotl, zebrafish) might lead to novel strategies in the field of mammalian stem cells. For instance, stimulation of endogenous neurogenesis upon diseases or trauma could be a simple, but an effective way, for cell replacement. To investigate such issues, the spinal cord regeneration of tailed amphibians (e.g. axolotl) upon physical amputation became an established model of epimorphic regeneration [80]. Although, the general morphological aspects of this process were described several decades ago [18], [21], the fine details underlying the molecular machinery are only in the beginning to be understood [81]. Moreover, there are still uncertainties about the cellular rearrangement of constituents that are involved, and it is still an open question whether a pre-existing, minute population of stem cells or de-differentiating cells could be the primary sources of regeneration [82].

By documenting expression of prominin-1 in normal versus regenerative spinal neural tube of axolotl, we could demonstrate that prominin-1–positive cells significantly expand from their restricted localization in the floor plate toward more dorsal co-ordinates in the regenerating neural tube. Such a situation is strikingly reminiscent to what has been observed for sonic hedgehog (Shh) both in axolotl and zebrafish in response to injury [30], [81]. These findings argue for the importance of the floor plate as well as the expanded Shh-signal emitting cells in co-ordinating the spinal cord tissue repair, beyond the well-known function of the midline floor in elaborating positional identity along the dorso-ventral axis of the spinal cord in vertebrates [83]. Although, recent investigation of a tumor model system further exemplifies a likely relation between the Shh-pathway and prominin-1–positive cells [84], to uncover the exact details of that in the neural tube requires further examination.

In axolotl, the induced prominin-1–positive cells that are lining the central canal might correspond to the activated ependymal cell populations that are the major source of post-mitotic neurons in the regenerating spinal cord [82], [85], [86]. Such observations also strengthen the similarities between neurogenic progenitor cells of regenerating non-mammalian spinal neural tube and developing mammalian CNS [reviewed in 22]. Whether prominin-1–positive ependymal cells of the normal, intact axolotl spinal neural tube are per definition stem cells (e.g. in dormant state) is not clear yet [28], [87]. Nevertheless, the cell surface appearance of prominin-1 might help their isolation for further characterization both at molecular (e.g. transcriptome) and cellular (e.g. transplantation assay) levels. Such investigation should bring novel facets of regeneration.

### Prominin's Function and Gene Products

Although the physiological function of prominin-1 is not yet resolved, its expression in various neurogenic zones among distinct species (this study) as well as in somatic stem cells originating from diverse mammalian organs is very suggestive for a role either in maintaining stem and progenitor properties or proliferative abilities [reviewed in 88]. This notion is further supported by existence of prominin-1–positive cancer stem cells such as those derived from gliomas [89]. The asymmetric/symmetric distribution of prominin-1 among descendants of mitotic neural and hematopoietic stem cells or cancer stem cells derived thereof [88], [90]–[92], and its release by means of small membrane vesicles into extracellular milieu is somehow related to the switch of proliferation process to cellular differentiation [73], [93], [94]. However, it should be kept in mind that the widespread expression of prominin-1 in various other terminally differentiated somatic cell types (e.g. differentiated epithelial and glial cells, photoreceptors) is very suggestive of a function that is beyond the control of stem cells [35], [45], [95]–[98]. Prominin-1 as a cholesterol-binding membrane protein associated with specific membrane microdomains may act as an organizer of the plasma membrane of protrusions where it is preferentially concentrated [reviewed in 43,99]. Suggestive of multiple cellular roles and/or a sub-functionalization of prominin-1 is the existence of numerous splice variants [100], [101], which are developmentally regulated as demonstrated recently in the murine brain [45], and the differential expression pattern of prominin-1a and prominin-1b observed here in the adult zebrafish brain. It remains to be determined whether the overall expression pattern of prominin-1 molecules in non-mammalian vertebrates relies solely on a given splice variant or distinct prominin-1 isoforms. Nevertheless, the acquired information offer several loss-of-function and gain-of-function approaches in experimental organisms that might point out the physiological implication(s) of prominin-1 in a given tissue or organ.

## Conclusion

The present study reveals that the tissue localization of prominin-1–positive cells in the non-mammalian vertebrate CNS is markedly reminiscent to that in mammals. Thus, prominin-1 expression highlights neurogenic germinative zones within the intact brain of various species, and suggests as well an involvement of prominin-1–positive cells in provoked, compensatory neurogenesis. Its use as a marker of stem cells in conjunction with other cell surface antigens – in a positive or negative selection paradigm – might help the identification of putative neural stem cells. One always should be aware about potential context dependent, often dual (neurogenic and non-neurogenic), expression characteristics of stem- and progenitor markers. Collectively, the current data add an important asset to the toolbox for dissecting further interspecies conservation of neurogenic niches within the animal kingdom.

## Supporting Information

Figure S1 **Distribution of murine prominin-1–positive cells in the developing telencephalon.** (A–C) Cryosections of brains from mice at embryonic day 14 (E14) were processed for ISH using an antisense DIG-labelled prominin-1 probe. Cross-sections of a telencephalic hemisphere at the level of the interventricular foramen (A) and behind it (B), and close to the occipital pole (C) indicate expression of prominin-1 (black arrowheads) in the ventricular zone of the dorsal/medial, dorsal and caudal pallium (A, B, C, respectively). Prominin-1–positive cells are detected also in the subpallial ganglionic eminence (B: blue arrowhead). Note that the mantle zone (A, C; black hollow arrowheads) and the invaginating choroid plexus (B; cp) are devoid of prominin-1. LV, lateral ventricle. Scale bars, A–C, 100 µm.
(TIFF)

Figure S2 **Differential expression of zebrafish prominin-1a and b in the prosencephalon.** (a–c') Higher magnification of insets displayed in panels A–C of Figure 6. Proliferative zones are indicated with numbers (see legend of figure 6). Coloured arrows and asterisks indicate overlapping expression domains of particular genes. (a, a') Prominin-1a is strongly expressed in proliferating zones of the ventral telencephalon (vT) encompassing the rostral migratory stream-like assembly (RMS), preoptic area (PO), periventricular hypothalamus (PVH) including areas surrounding the posterior recessus (PR). Extending into the extraventricular, prominin-1a is detected in the diffuse nucleus of the hypothalamic inferior lobe (DIL). (b, b') In contrast to prominin-1a, prominin-1b is excluded from the most extensively proliferating subdivisions of the prosencephalon (brown arrows). It is only weakly expressed in smaller subdomains of the vT, PO and PVH. (c, c') Like prominin-1a, msi1 is detected in all of the extensively proliferating zones of the telencephalon (vT) and diencephalon (PO, PVH, posterior tuberculum (PT), PR). Cant, commissura anterior; CM, corpus mamillare; DiV, diencephalic ventricle; Ha, habenula; OB, bulbus olfactorius; SD, saccus dorsalis. Scale bars, a–c', 100 µm.
(TIFF)

Figure S3 **The combined expression of prominin-1a and b mimics distribution of musashi-1 in adult zebrafish brain.** (A–C) Cryosections of 3-month-old adult brain from BrdU-treated zebrafish were processed for ISH using an antisense DIG-labelled probe either against prominin-1a (A; *prom1a*), prominin-1b (B; *prom1*b) or musashi-1 (C; *msi1*). Proliferating cells were observed by immuno-detection of BrdU (brown). Position of paramedian sections of the brain is indicated on the cartoon (A) adapted from a standard neuroanatomical atlas by Wulliman and colleagues [104]. Coloured arrows indicate overlapping expression domains of particular genes. Black dashed line indicates the border of prosencephalon towards the tegmentum of the brainstem (A). Major sites of prominin-1a (A) expression are located in the prosencephalic and tectal domains overlapping partly with msi1 (C) in certain subdivisions of the dorsal telencephalic surface proliferative zone (3), in a subdivision of the preoptic area (PO) located above the optic tract (OT), in the periventricular hypothalamus (PVH), in the periventricular grey zone (PGZ) and torus longitudinalis (TL). Expression of prominin-1a extends laterally into msi1–negative areas including the extraventricular parenchyma of the dorsal telencephalon (dT, asterisk), central parts of the ventral telencephalic area (Vc) and diffuse nucleus of the hypothalamic inferior lobe (DIL). Prominin-1b (B) overlaps with msi1 (C) in the periventricular rhombencephalon (*griseum centrale*, GC) and valvula cerebelli (Va). All three genes are detected in the dorsal thalamus (DT) and in the facial lobe (LVII). CC, crista cerebellaris; CCe, Corpus cerebelli; CM, corpus mamillare; Ha, habenula; LX, vagal lobe; OB, olfactory bulb; PR, posterior recessus of the diencephalic ventricle; VT, ventral thalamus. Scale bars, A–C, 250 µm.
(TIFF)
