# Supplementary figures and images for: Spatial Distribution of Prominin-1 (CD133) – Positive Cells within Germinative Zones of the Vertebrate Brain
Source: PLoS One. 2013 May 27;8(5):e63457. doi: 10.1371/journal.pone.0063457 (PMC3664558; doi:10.1371/journal.pone.0063457)

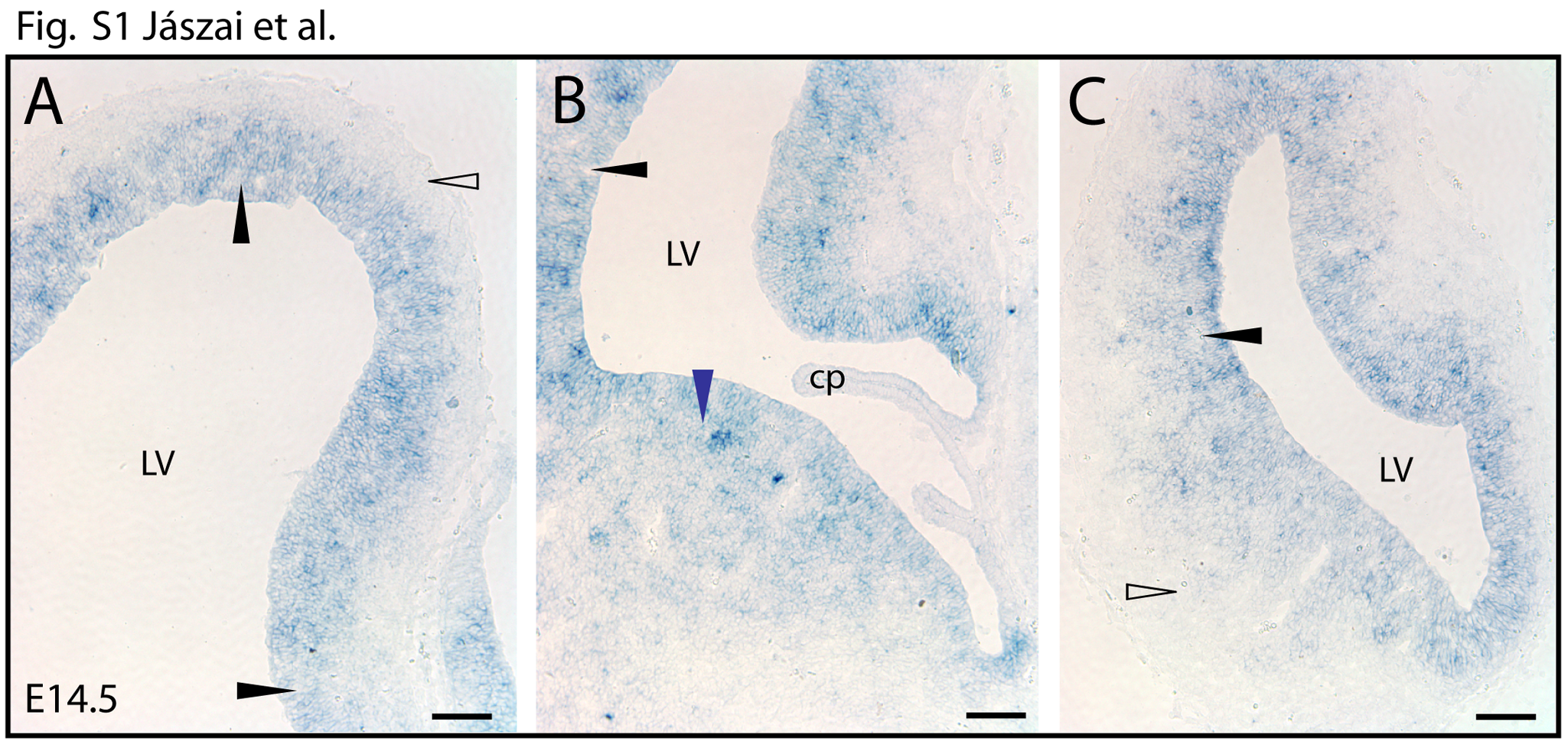

Supplement: Figure S1 — Distribution of murine prominin-1–positive cells in the developing telencephalon. (A–C) Cryosections of brains from mice at embryonic day 14 (E14) were processed for ISH using an antisense DIG-labelled prominin-1 probe. Cross-sections of a telencephalic hemisphere at the level of the interventricular foramen (A) and behind it (B), and close to the occipital pole (C) indicate expression of prominin-1 (black arrowheads) in the ventricular zone of the dorsal/medial, dorsal and caudal pallium (A, B, C, respectively). Prominin-1–positive cells are detected also in the subpallial ganglionic eminence (B: blue arrowhead). Note that the mantle zone (A, C; black hollow arrowheads) and the invaginating choroid plexus (B; cp) are devoid of prominin-1. LV, lateral ventricle. Scale bars, A–C, 100 µm. (TIFF) [file pone.0063457.s001.tiff]

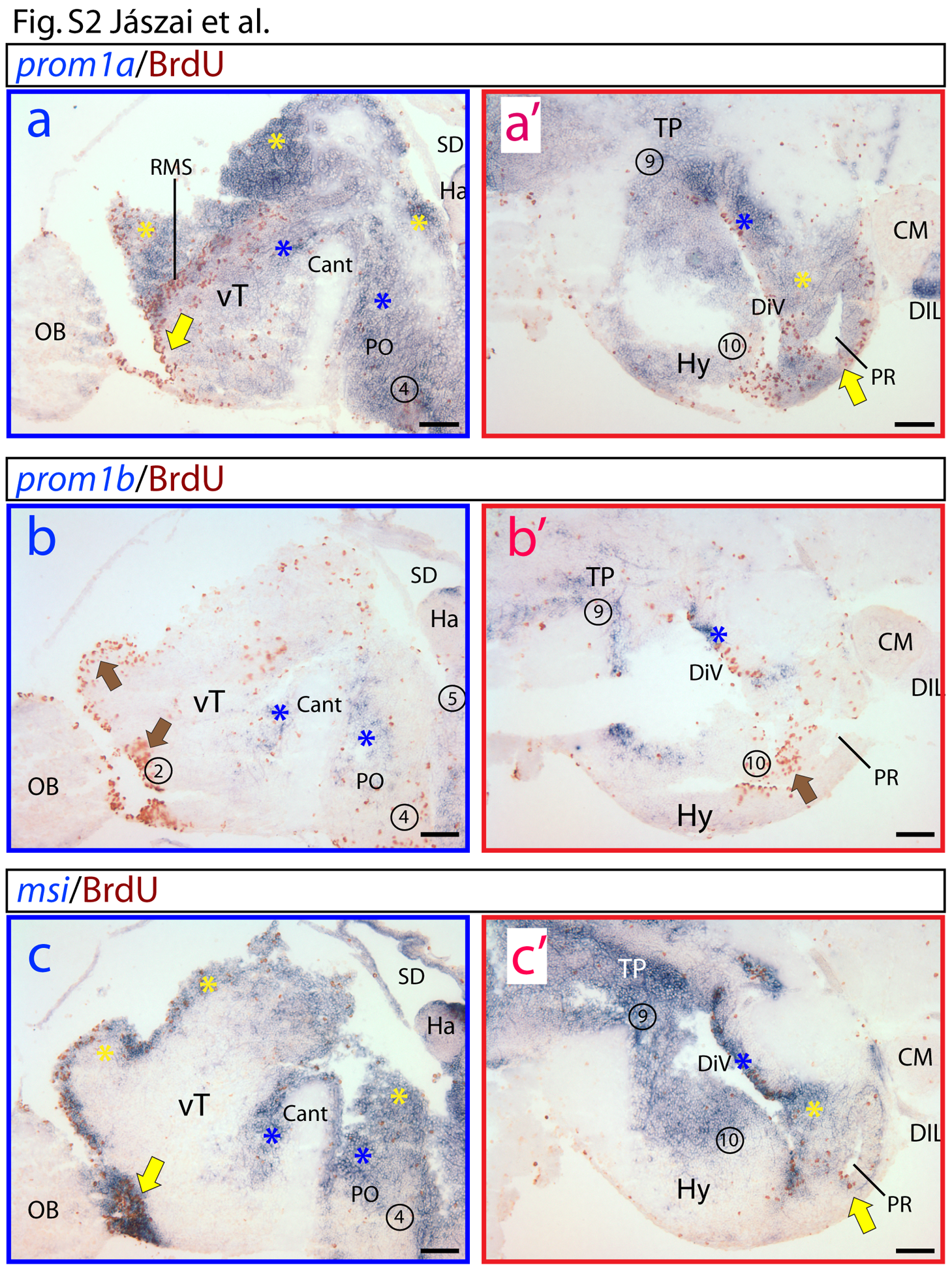

Supplement: Figure S2 — Differential expression of zebrafish prominin-1a and b in the prosencephalon. (a–c') Higher magnification of insets displayed in panels A–C of Figure 6. Proliferative zones are indicated with numbers (see legend of figure 6). Coloured arrows and asterisks indicate overlapping expression domains of particular genes. (a, a') Prominin-1a is strongly expressed in proliferating zones of the ventral telencephalon (vT) encompassing the rostral migratory stream-like assembly (RMS), preoptic area (PO), periventricular hypothalamus (PVH) including areas surrounding the posterior recessus (PR). Extending into the extraventricular, prominin-1a is detected in the diffuse nucleus of the hypothalamic inferior lobe (DIL). (b, b') In contrast to prominin-1a, prominin-1b is excluded from the most extensively proliferating subdivisions of the prosencephalon (brown arrows). It is only weakly expressed in smaller subdomains of the vT, PO and PVH. (c, c') Like prominin-1a, msi1 is detected in all of the extensively proliferating zones of the telencephalon (vT) and diencephalon (PO, PVH, posterior tuberculum (PT), PR). Cant, commissura anterior; CM, corpus mamillare; DiV, diencephalic ventricle; Ha, habenula; OB, bulbus olfactorius; SD, saccus dorsalis. Scale bars, a–c', 100 µm. (TIFF) [file pone.0063457.s002.tiff]

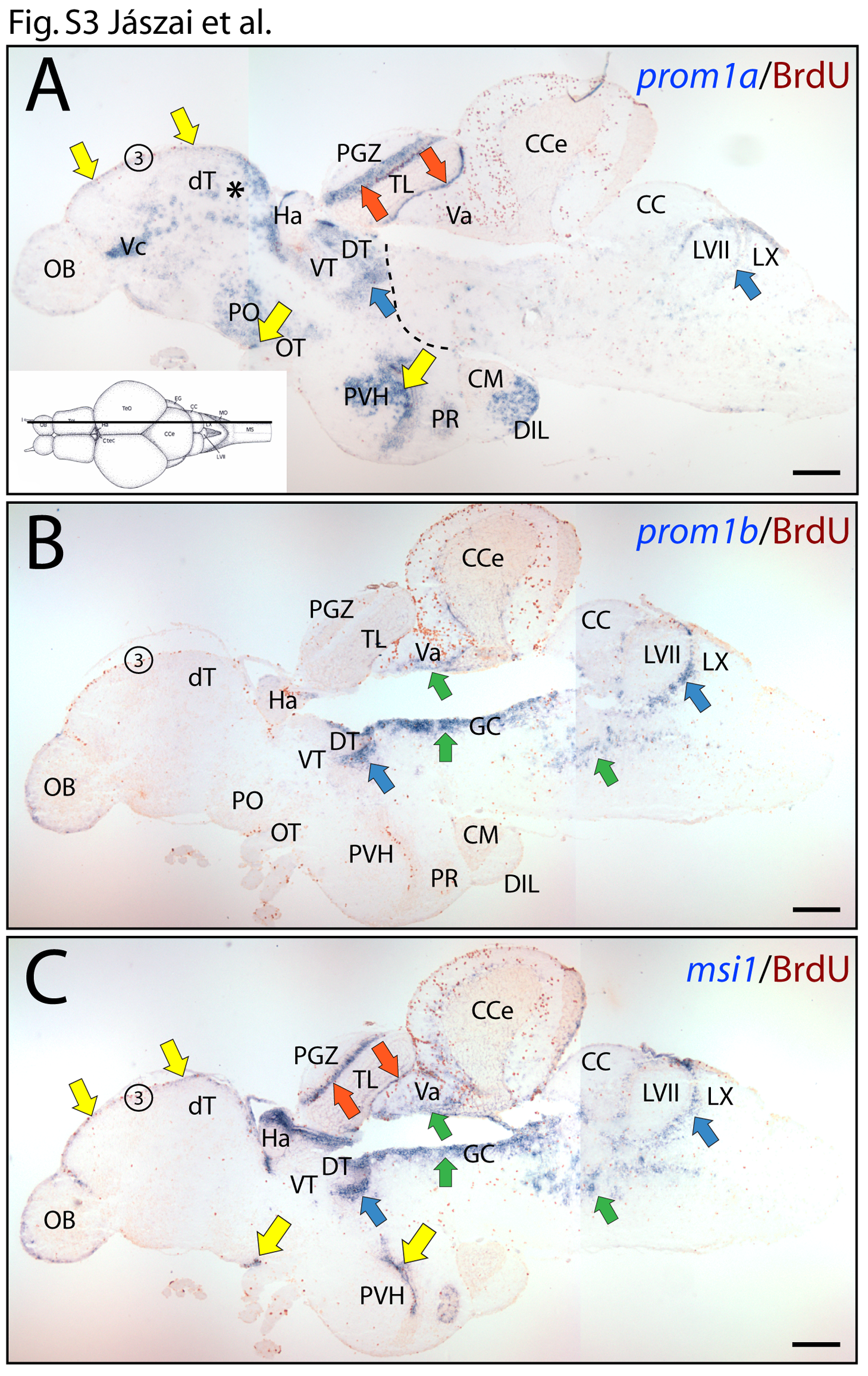

Supplement: Figure S3 — The combined expression of prominin-1a and b mimics distribution of musashi-1 in adult zebrafish brain. (A–C) Cryosections of 3-month-old adult brain from BrdU-treated zebrafish were processed for ISH using an antisense DIG-labelled probe either against prominin-1a (A; prom1a), prominin-1b (B; prom1b) or musashi-1 (C; msi1). Proliferating cells were observed by immuno-detection of BrdU (brown). Position of paramedian sections of the brain is indicated on the cartoon (A) adapted from a standard neuroanatomical atlas by Wulliman and colleagues [104]. Coloured arrows indicate overlapping expression domains of particular genes. Black dashed line indicates the border of prosencephalon towards the tegmentum of the brainstem (A). Major sites of prominin-1a (A) expression are located in the prosencephalic and tectal domains overlapping partly with msi1 (C) in certain subdivisions of the dorsal telencephalic surface proliferative zone (3), in a subdivision of the preoptic area (PO) located above the optic tract (OT), in the periventricular hypothalamus (PVH), in the periventricular grey zone (PGZ) and torus longitudinalis (TL). Expression of prominin-1a extends laterally into msi1–negative areas including the extraventricular parenchyma of the dorsal telencephalon (dT, asterisk), central parts of the ventral telencephalic area (Vc) and diffuse nucleus of the hypothalamic inferior lobe (DIL). Prominin-1b (B) overlaps with msi1 (C) in the periventricular rhombencephalon (griseum centrale, GC) and valvula cerebelli (Va). All three genes are detected in the dorsal thalamus (DT) and in the facial lobe (LVII). CC, crista cerebellaris; CCe, Corpus cerebelli; CM, corpus mamillare; Ha, habenula; LX, vagal lobe; OB, olfactory bulb; PR, posterior recessus of the diencephalic ventricle; VT, ventral thalamus. Scale bars, A–C, 250 µm. (TIFF) [file pone.0063457.s003.tiff]
